# WASP: Wearable Analytical Skin Probe for Dynamic Monitoring of Transepidermal Water Loss

**DOI:** 10.1021/acssensors.3c01936

**Published:** 2023-11-13

**Authors:** Anjali Devi Sivakumar, Ruchi Sharma, Chandrakalavathi Thota, Ding Ding, Xudong Fan

**Affiliations:** † Department of Biomedical Engineering, University of Michigan, Ann Arbor, Michigan 48109, United States; ‡ Department of Electrical Engineering and Computer Science, University of Michigan, Ann Arbor, Michigan 48109, United States; § Center for Wireless Integrated MicroSensing and Systems (WIMS2), University of Michigan, Ann Arbor, Michigan 48109, United States; ∥ Max Harry Weil Institute for Critical Care Research and Innovation, University of Michigan, Ann Arbor, Michigan 48109, United States

**Keywords:** transepidermal water loss, skin barrier, stratum corneum, Fick’s laws of diffusion, evaporative flux, sweat rate

## Abstract

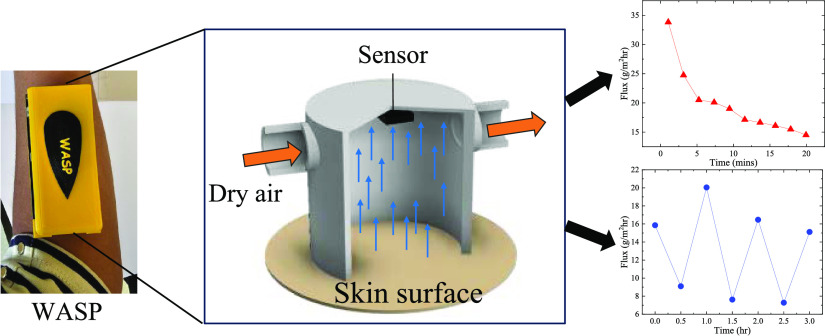

Early diagnosis of skin barrier dysfunction helps provide timely preventive care against diseases such as atopic dermatitis, psoriasis, food allergies, and other atopic skin disorders. Skin barrier function is commonly evaluated by measuring the transepidermal water loss (TEWL) through stratum corneum due to its noninvasive characteristics. However, existing commercial TEWL devices are significantly affected by many factors, such as ambient temperature, humidity, air flow, water accumulation, initial water contents on the skin surface, bulky sizes, high costs, and requirements for well-controlled environments. Here, we developed a wearable closed-chamber hygrometer-based TEWL device (Wearable Analytical Skin Probe, WASP) and the related algorithm for accurate and continuous monitoring of skin water vapor flux. The WASP uses short dry air purges to dry the skin surface and chamber before each water vapor flux measurement. Its design ensures a highly controlled local environment, such as consistent initial dry conditions for the skin surface and the chamber. We further applied WASP to measure the water vapor flux from six different locations of a small group of human participants. It is found that the WASP can not only measure and distinguish between insensible sweating (*i.e*., TEWL) and sensible sweating (*i.e*., thermal sweating) but also track skin dehydration–rehydration cycles. Comparisons with a commercial TEWL device, AquaFlux, show that the results obtained by both devices agree well. The WASP will be broadly applicable to clinical, cosmetic, and biomedical research.

Recent advancements in skin barrier research have brought to light the intricate mechanisms underlying various common skin diseases.^1,2^ This significant progress has been made following the discovery of the filaggrin mutation (FLG) in patients with atopic dermatitis (AD).^3^ FLG, a critical epidermal protein essential for skin barrier formation, represents a major risk factor for AD. Moreover, investigations have revealed a correlation between the FLG mutation and conditions such as asthma and food allergies, even in the absence of AD.^4^ Additionally, a recent study has highlighted the significant potential of skin barrier integrity as a valuable biomarker for the early detection of life-threatening food anaphylaxis, enabling timely intervention before symptom onset.^5,6^

Apart from the impaired skin barrier associated with common atopic skin disease conditions, climatic conditions, particularly during winter, can further exacerbate skin barrier dysfunction due to decreased levels of lignoceric and heptadecanoic acids.^7^ Additionally, chronic exposure to air pollution plays an important role in disrupting the skin barrier.^8^ Air pollution and particulate matter have the potential to cause substantial damage to the protective epithelial barrier by inducing oxidative stress through reactive oxygen species.^9^ This oxidative barrier disruption, in turn, can aggravate dermatologic conditions like AD and trigger immune system activation cascades.^10^ Hence, early identification of skin barrier dysfunction in individuals affected by these factors is essential for timely preventive care interventions.

Different skin analysis methods,^11^ like transepidermal water loss (TEWL) measurement, which is also known as insensible sweating rate measurement,^12−15^ Raman spectroscopy,^16−18^ and imaging techniques such as optical coherence tomography^19−21^ and laser scanning microscopy,^22,23^ have been developed over the years to monitor skin barrier integrity. Owing to its noninvasive and cost-effective nature, TEWL is most widely used as a parameter for evaluating skin barrier function compared to the other skin barrier analysis methods.^24−26^ It is known that the entire body can produce both sensible sweat (through sweat glands due to external stimuli, such as heat) and insensible sweat without any external stimulus. Insensible sweating results from water within the body osmotically diffusing and unconsciously evaporating from the inner dermis and epidermis to the outermost layer of the skin called the stratum corneum (SC), driven by a water gradient. Most insensible sweat evaporates from the skin surface into the surrounding environment, which is TEWL, while a portion is retained within the SC to maintain skin hydration.^27,28^ In healthy skin, efficient moisture retention leads to normal TEWL values (∼5–40 g/m^2^h, depending on body locations and ages), whereas high or low TEWL values indicate skin barrier dysfunction or an intact/recovered skin barrier, respectively.

Over the past two decades, various commercial hygrometer-based TEWL measurement devices have been developed for clinical and cosmetic applications, including, for example, Tewameter,^13^ GPSKIN,^15^ Vapometer,^14^ AquaFlux,^12^ and DermaLab.^29^ All of these commercial devices are configured in an open- or closed-chamber format to estimate the TEWL values by analyzing the microclimate created by the diffusive water vapor flux from the skin. As summarized in Table S1, the open-chamber TEWL devices are susceptible to environmental factors, such as ambient temperature and humidity, and air flow. Consequently, the examinees are required to wait in the test environment, where temperature and humidity (and possibly air flow) are controlled for a certain period of time (∼20 min) before measurement.^24,25^ No motion is allowed during the measurement. On the other hand, the closed-chamber TEWL devices may encounter a problem of water accumulation inside the chamber. Furthermore, the initial water content on the skin surface and initial humidity inside the chamber may affect the TEWL measurement for some closed-chamber TEWL devices.^14^ Finally, nearly all commercial TEWL devices are bulky and cannot be made wearable.

Recently, there have also been strides toward developing wearable TEWL devices (or more generally speaking, wearable sweat analysis devices that measure the combination of sensible and insensible sweating) that operate on various mechanisms,^27^ including hygrometer-based,^30−33^ absorbent-material-based,^34−37^ and microfluidics-based^38−42^ principles (see the summary in Table S2). However, these wearable devices still encounter issues similar to the commercial ones (*i.e*., environmental changes, initial water contents on the skin surface, and initial humidity inside the chamber, and/or, water accumulation, *etc*.). An ideal TEWL (or sweating rate analysis) device would be wearable, independent of ambient factors (such as temperature, humidity, and air flow), independent of initial conditions (such as the water content on the skin surface and initial humidity inside the measurement chamber), and able to monitor water vapor flux accurately and continuously. It is also highly desired if the device can distinguish between the sensible and insensible sweating; since most of the wearable devices provide only the total sweating rate, nearly all commercial devices require a controlled, relatively low temperature so that the sensible sweating is significantly suppressed.

Here, we developed and fabricated a wearable, hygrometer-based, closed-chamber TEWL device that can provide continuous skin water vapor flux measurement without interference from the environment and skin and chamber initial conditions. Our TEWL device is conceptually illustrated in Figure 1(A). It consists of a hollow chamber with one end open to the skin to be analyzed and the other end closed. Two fluidic inlet/outlet channels are added to pump dry air into the chamber to flush out the accumulated water vapor from the chamber and remove residual water on the skin surface before each measurement. The dry air can be provided by an external source (such as a dry air cylinder) or an internal source. The internal source design is particularly suitable for a wearable device in which dry air circulates internally by a pump and a moisture filter set installed in the wearable device. The relative humidity and temperature (RH-T) transients inside the chamber due to the water vapor flux diffused from the skin are monitored with an RH-T sensor placed on the ceiling (for instance, 4.75 mm from the skin surface in our device) of the chamber. The water vapor flux values are then extracted from these sensor readings using Fick’s first and second laws of diffusion,^43^ in combination with our algorithm described in the following sections.

**Figure 1:**
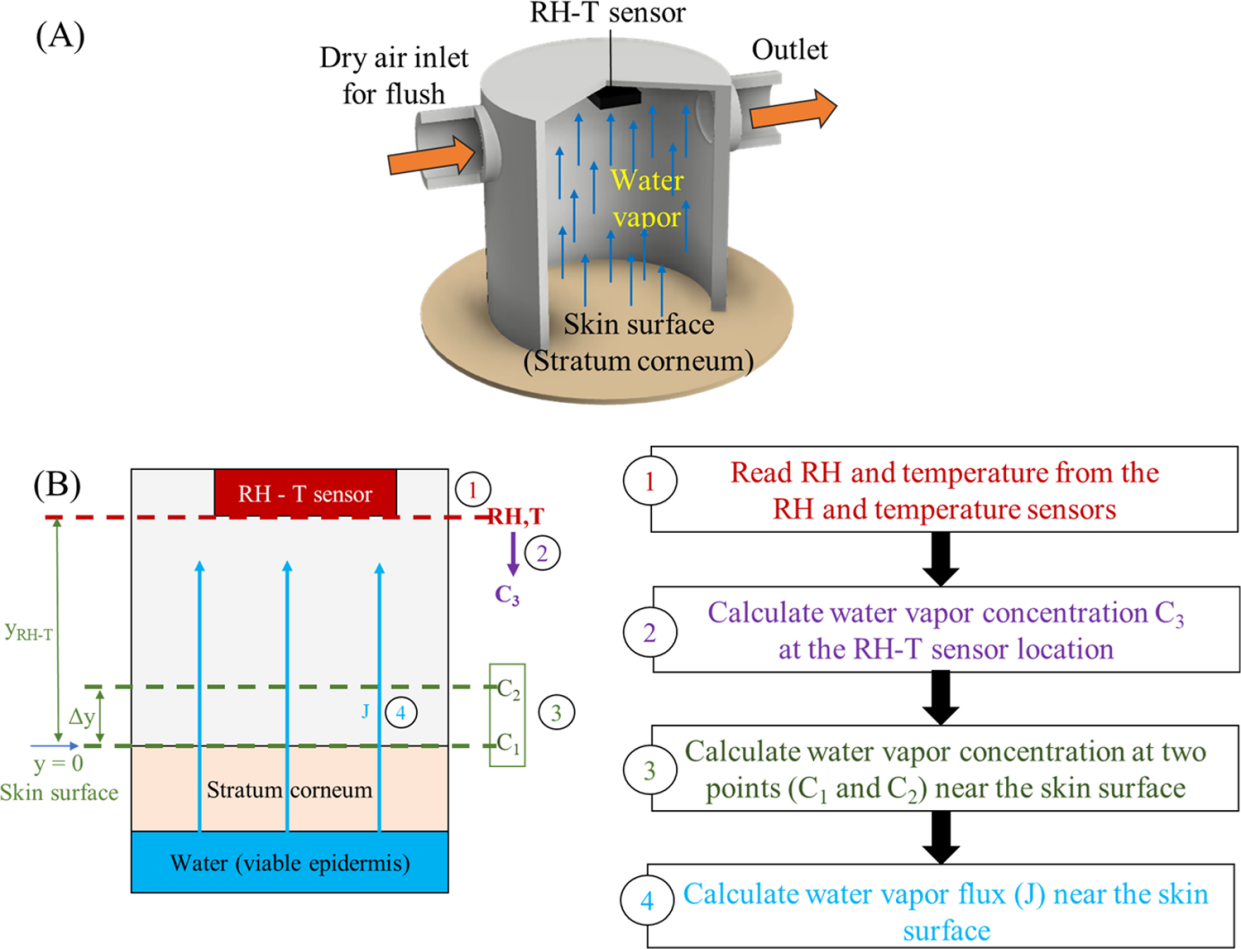
(A) Schematic of our closed-chamber TEWL device design. Initially, dry air is used to purge the moisture inside the chamber and residual water on the skin surface. Then, the temporal response of the relative humidity-temperature (RH-T) sensor is measured right after the purge to calculate the TEWL using a mathematical model. The above process, *i.e*., purge/measure, can be repeated indefinitely. (B) Protocol for water vapor flux calculation.

There are a few distinct advantages of the TEWL device. First, the closed-chamber design ensures that the measurement is not affected by the surrounding environment, such as ambient temperature/humidity and air flow. Second, dry air flush ensures identical initial conditions, *i.e*., dry skin surface and nearly 0% RH level inside the chamber for all water vapor flux measurements. Third, as shown later, the final device weighs only about 65 g and is wearable. Finally, the device can be used to continuously monitor the sweating rate, distinguish sensible sweating and insensible sweating (*i.e*., TEWL), and measure skin dehydration–rehydration cycles, all of which are difficult to accomplish with all other devices.

## Mathematical Modeling

Unlike the open-chamber and condenser-based approaches, in our method, the water vapor concentration and hence the concentration gradient and evaporative water vapor flux in the chamber change over time because of water vapor accumulation during the water vapor flux measurement period. This process, as illustrated in Figure 1(B), can be modeled using one-dimensional Fick’s second law of diffusion,^43^*i.e*.
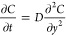
1where *C* is the concentration of water vapor at any point, *y*, along the chamber height at time *t*. *D* is the diffusion coefficient of water vapor in the air inside the chamber. Here, we assume that there is no convection inside the chamber, and the water vapor motion is caused only by diffusion. In our model, we use the following boundary and initial conditions.

1. *C* (*y* > 0, *t* = 0) = *C*_0_. C_0_ = Initial concentration of water vapor in the chamber.
2. *C* (*y* = 0, *t* > 0) = *C*_s_. Here, we assume that the water source (the skin) is a nondepleting source that provides constant water vapor concentration *C*_s_ on the skin surface.
3. *C* (*y* = ∞, *t* = 0) = 0. Here, we assume that the length of the chamber is infinite, although a closed chamber with a finite height (or volume) was used in our actual device. This assumption significantly simplifies the mathematical modeling, as the concentration at any spatial point at a given time can be analytically calculated (see eq 2 below). The validity of using this open-chamber mathematical model for our closed chamber to estimate the water vapor flux is discussed in S1.1 of the Supporting Information.

Solving eq 1 using the above boundary and initial conditions yields^44^
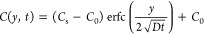
2Equation 2 can thus be used to calculate the concentration transient of water vapor at any spatial point along the chamber height in the chamber if the water vapor concentration at any other spatial point at a given time is known. Therefore, using eq 3, the water vapor concentration at the skin surface (*C*_s_) can be calculated from *C*_3_ obtained by the RH-T sensor (placed at a distance of *y*_RH-*T*_ from the skin surface). This *C*_s_ value can later be used to calculate the water vapor concentration at two spatial points very close to the skin surface (*i.e*., *C*_1_ and *C*_2_ in Figure 1(B)) by using eq 2
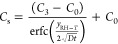
3Finally, Fick’s first law (eq 4) is used to calculate the water vapor flux from the calculated water vapor concentrations at two spatial points near the skin, *i.e*.,
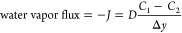
4The overall process is illustrated in Figure 1(B).

Practically, *C*_0_, the initial water vapor concentration inside the chamber, is obtained by the RH sensor just before the measurement starts. It consistently decreases down to ∼5% after purge. The spatial point for *C*_1_ is chosen to be *y* = 0, *i.e*., the skin surface. The detailed description of how to choose the spatial point for *C*_2_ (*y* = 2 mm in our case, so that Δ*y* = 2 mm) is presented in S1.2 in the Supporting Information. It should be noted that in the above model, we assume that the humidity sensor response is instantaneous. Practically, the humidity sensor has a finite response time. S1.3 and Figure S3 in the Supporting Information show how the sensor response delay may affect the water vapor flux peak value and the time when the flux peak value is reached.

## Materials and Methods

**Figure 2:**
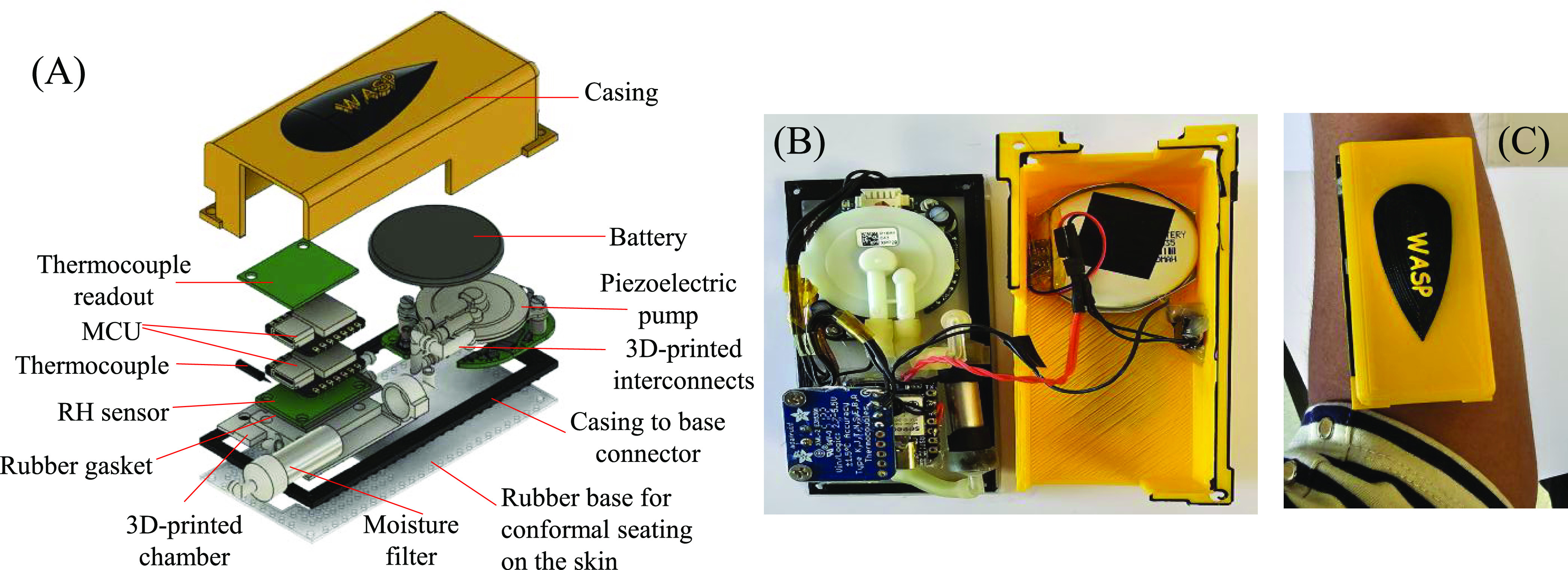
Our Wearable TEWL Device―Wearable Analyzer for Skin Porosity (WASP): (A) exploded view, (B) interior view of the assembled device, and (C) WASP mounted on the forearm of a subject using a 3M double-sided adhesive tape.

In this work, we constructed two types of water vapor flux measurement devices. The first one was the “TEWL module”, which was used for characterization experiments. The details of the TEWL module design, fabrication, and assembly are presented in S2. The second one was the “Wearable Analytical Skin Probe (WASP)”, which was fully automated and wearable and used for skin water vapor flux measurement on human subjects (see Figure 2). The chamber, RH sensor, and thermocouple used in the WASP were adapted from the TEWL module. To achieve internal dry air circulation within the chamber, we integrated a piezoelectric pump P/N: UXPC5400200A, LEE Company, and a moisture filter consisting of a lightweight aluminum tube (3.5 mm in diameter and 30 mm in length, P/N: 9806, K&S Precision Metal) containing Molecular Sieve 5A (P/N: 20302, Supelco) as the desiccant with glass wool plugs (P/N: 20411, Supelco). Although more expensive than other pumps, the pump that we selected for the WASP has low noise and vibration characteristics, thus ensuring accurate water vapor flux measurements and user comfort. A rechargeable 3.85 V, 450 mAh Li polymer battery (P/N: 403535, AliExpress) was used to power the WASP. Device control and data collection were managed by two microcontroller units (MCUs) that transmitted data to a laptop via Bluetooth. The WASP’s main casing was 3D-printed using a PLA filament (Ultimaker). The rubber base holder, which forms the supportive framework for the TEWL chamber and pump, was 3D-printed using a flexible material (Flexible 80A resin (P/N: RS-F2-FL80-01, Formlabs)) to maximize user comfort and ensure conformal contact with the skin. The bill of materials is given in Table S3.

The WASP had a weight of 65 g and exterior dimensions of 89.9 × 40.9 × 28 mm. The moisture filter and battery both could last for 4 h of continuous operation (or ∼90 runs) even with bluetooth turned on. During the human subject testing, the WASP was mounted firmly onto the skin surface using a disposable, medical-grade double-sided adhesive film (P/N: 1577, 3M Medical Materials and Technologies) to provide a good seal of the WASP with the skin and minimize the pressure applied to the skin.

### Operation Procedures

Both the nonwearable TEWL module and wearable WASP were operated according to the same procedures. Each flux measurement cycle was comprised of 60 s of dry air/N_2_ flush at a rate of ∼90 mL/min, followed by 60 s of RH-T sensor readings with no dry air/N_2_ flush. The measurement cycle can be repeated with any time interval in between. For example, a new measurement cycle can start right after the end of the 60 s RH-T reading or wait for 30 min for skin rehydration (when the purging air was kept off) or for skin dehydration (when the purging air was kept on).

### Water Vapor Flux Analysis Pipeline

**Figure 3:**
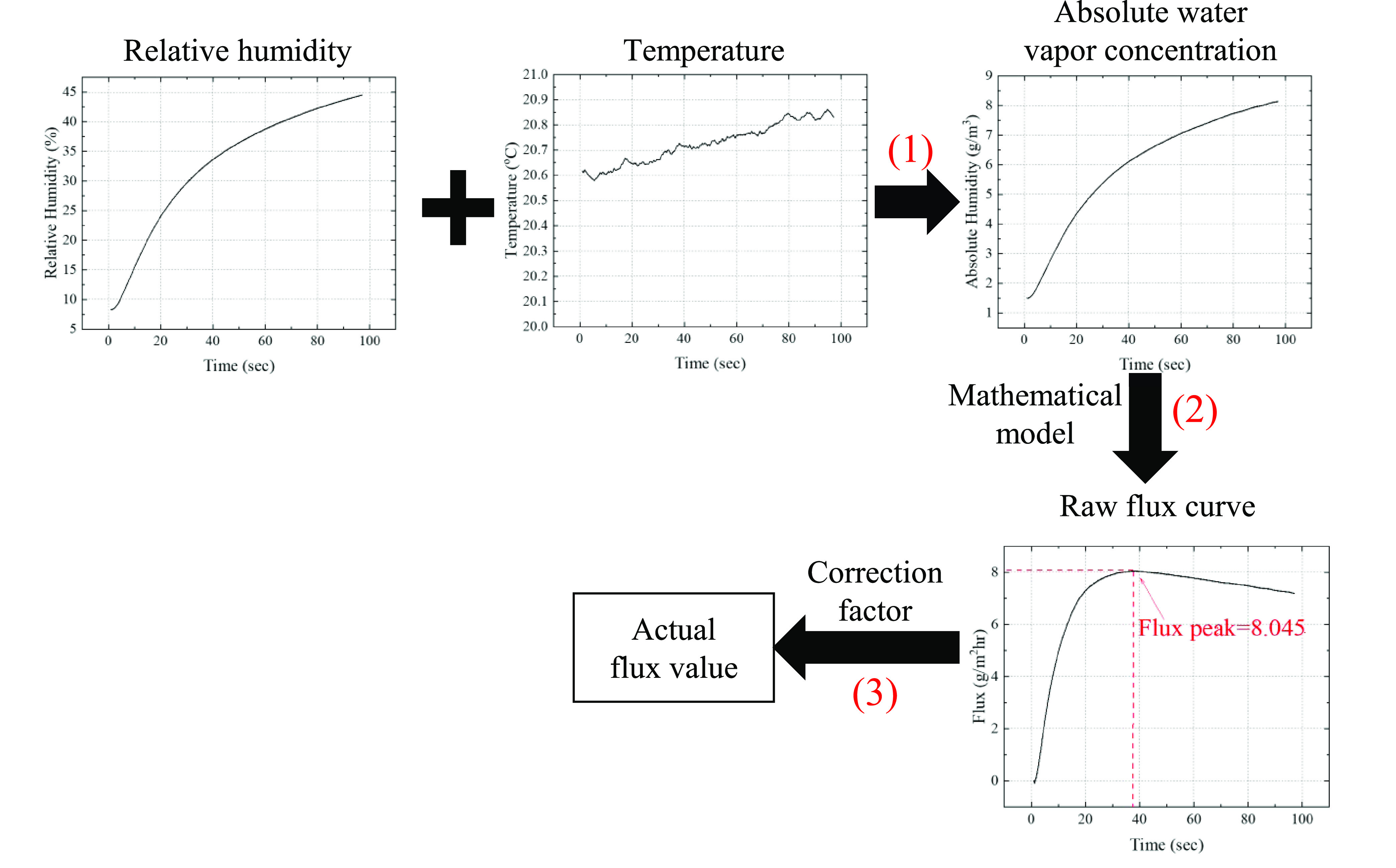
Process flow to obtain the actual water vapor flux value. (1) RH and temperature readings are combined to produce the absolute water vapor concentration curve as a function of time. (2) Mathematical model is applied to generate the water vapor flux curve. The maximal flux value and the corresponding time are recorded. (3) Correction factor is applied to the maximal flux obtained in (2) to produce the actual flux value. The curves in the panels are actual measurement data to show how the curve in each step looks like.

An in-house water vapor flux analysis pipeline, as depicted in Figure 3, was developed. First, the RH-T temporal readings are used to calculate the corresponding absolute water vapor concentration (or absolute humidity), which is C_3_ in Figure 1(B). Second, the temporal water vapor flux curve is calculated using the mathematical model detailed in the “Mathematical Modeling” section. Third, the maximal water vapor flux and the corresponding time were recorded. Finally, a correction factor was used to account for any nonidealities (such as the nonlinear time constant of the RH sensor) that will be discussed in the following section.

### Water Vapor Flux Correction Factor

**Figure 4:**
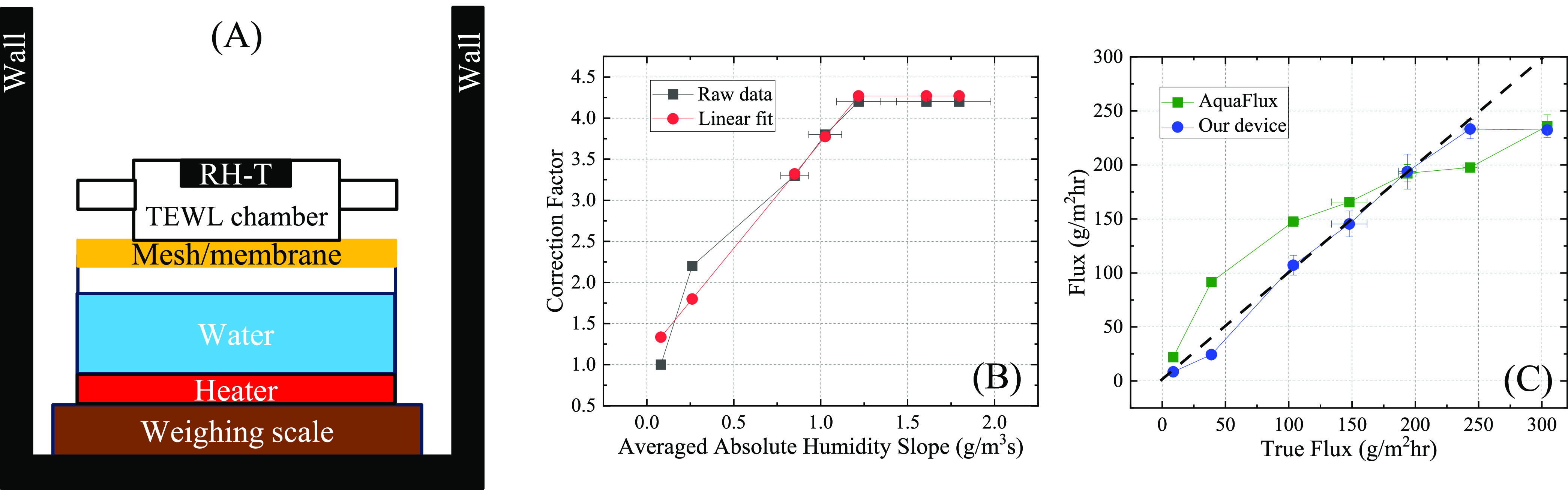
Correction factor curves. (A) Schematic of the wet-cup setup to generate different water vapor flux values. (B) Correction factor vs averaged absolute water vapor concentration (or humidity) slope measured by our device. The red curve is the linear fit up to 1.2 g/m^3^s. Error bars are obtained from at least 10 measurements. (C) Comparison of flux measurements from three different devices―weighing scale (true flux), our device (with a correction factor given by the red curve in panel (B)), and AquaFlux (error bars are obtained from at least five measurements). The black dashed line shows a perfect match between the measured flux values and the true flux to guide an eye.

A standard wet-cup method^14,31^ with artificial skin was used to generate known flux values and obtain the correction factor (Figure 4(A)). To ensure precise measurements across a wide flux range, we utilized two distinct wet-cup setups: wet-cup setup-1 (WCS1) and wet-cup setup-2 (WCS2). In WCS1, a polystyrene Petri dish (diameter: 54.4 mm) filled with water at room temperature (∼19 °C) was employed and the water evaporation rate (*i.e*., water vapor flux) was controlled by using two different types of semipermeable membranes, one with fine pores (OpSite Flexigrid, Smith and Nephew, England) and another one with coarse pores (304 Stainless Steel 150 Mesh, Uxcell). WCS2 employed a wireless mug with integrated heater (Vsitoo, Amazon) filled with water and covered by a semipermeable membrane (304 Stainless Steel 150 Mesh, Uxcell) (see Figure S5(A)). Water temperature was controlled to generate different evaporation rates. During the water vapor flux measurements with the WCS2, the initial 20 min of wait time was allotted after TEWL module is placed on the wet-cup setup to allow the chamber to reach thermal equilibrium with the wet cup before the start of water vapor flux measurement.

A weighing scale was used in both these setups to continuously measure the loss of water mass at intervals of approximately 3 s over a duration of at least 10 min. WCS1 employed a high-resolution weighing scale (P/N: USS-DBS83-120G, U.S. Solids) to accurately measure small water loss weights, whereas WCS2 used a larger-range weighing scale (P/N: JFDBS00058-500G, U.S. Solids). The periphery of the weighing scale and water cup was surrounded by a rigid plastic wall to avoid any measurement errors due to the convection of ambient air. The water vapor flux values were obtained by dividing the water mass loss by both the time and area of the Petri dish or mug. As a result of this comprehensive process, the wet-cup setups could generate seven flux data points ranging from 8 to 300 g/m^2^h.

## Results and Discussion

### Characterization of the RH Sensor and Temperature Sensor

Section S3 describes the RH sensor characterization. Overall, the RH sensor shows excellent repeatability (Figure S5(B)). However, the RH sensor exhibits a response time of approximately 16 s at moderate humidity, and the response time increases at elevated humidity levels. Based on the previous discussion that the sensor response time affects the flux measurement values, the nonlinear response time with respect to the humidity level calls for a correction factor for our devices.

Section S4 describes the temperature sensor characterization. It was found that the onboard SHTC3′s temperature sensor has a lower accuracy and longer response time than a thermocouple. Therefore, in our device design, we chose to use a Kapton capped K-type thermocouple. We further found that the Kapton tape used to cover the thermocouple measuring tip did not affect the temperature reading.

### RH Sensor Delay Compensation Curves

To meet the needs of dynamic RH measurement in our devices, it is crucial to employ an RH sensor with an instantaneous response, *i.e*., zero delay. But in practice, these sensors have a finite delay that affects the accuracy of the flux measurements (see S1.3 in the Supporting Information). The RH sensor (i.e., SHTC3) used in our devices is a simple capacitive sensor with a moisture-sensitive layer that operates on Fick’s law of diffusion. Its response time may be affected by the characteristics of the sensor, such as the plate structure, thickness of the moisture-sensitive layer, ambient temperature, humidity, *etc*. For example, when humid air comes into contact with a colder sensor surface, moisture condensation may occur, which may impact the RH sensor’s response time.^45,46^ The condensation becomes more severe during measurements at higher RH levels and is one of the primary causes for the nonlinear response time of the RH sensor (see S3.2 in the Supporting Information).

An in-house algorithm was developed to accommodate the aforementioned nonidealities in actual water vapor flux measurements. In this algorithm, a correction factor is obtained by dividing the true water vapor flux measured with the wet-cup experiment shown in Figure 4(A) by the maximal flux value in the raw flux curve obtained by our device according to the procedures illustrated in Figure 3. Figure 4(B) shows that the correction factor depends linearly on the absolute humidity slope (*i.e*., the water vapor concentration vs time) averaged at 2, 4, and 6 s before the flux apex time (see Figure 3 for illustration of a flux peak). This can be understood as follows. At a low water vapor flux, the flux curve obtained by our device increases very slowly. Therefore, the slow RH sensor response did not affect the flux measurement. Consequently, the correction factor is close to unity. At an increased water vapor flux, the flux curve obtained from our device increased more rapidly. Consequently, the slow RH sensor response has an increased impact on the flux measurement. Therefore, the correction factor becomes larger.

It should be noted that the correction factor curve exhibits linearity for the averaged absolute humidity slope below 1.2 g/m^3^s (corresponding to a true water vapor flux value of 200 g/m2 h), beyond which it levels off. This saturation phenomenon is indicative of reaching the upper limit of water vapor capacity that the designed chamber can hold within the measurement time (∼60 s) and the occurrence of water condensation. Depending on the application’s dynamic range, the chamber’s height can be increased, thereby augmenting its volume and capacity for water vapor.

Figure 4(C) plots the flux measured by our device against the true flux measured by the wet-cup method after the correction factor in Figure 4(B) is applied and shows good agreement. For comparison, parallel measurements were also performed by using an AquaFlux device (P/N: AF200, Biox Systems, U.K.) equipped with a reduced orifice cap (P/N: AF005-03, Biox Systems, U.K.) by holding it manually on top of the mesh/membrane in Figure 4(A). As shown in Figure 4(C), a noticeable discrepancy emerges between the flux measurements derived from the AquaFlux device and the true flux readings, which might be attributed to either external moisture infiltrating the measurement chamber (particularly noticeable in lower flux readings) or moisture escaping into the ambient surroundings (more pronounced with higher flux readings). This leakage issue is significantly mitigated in the case of our device as the double-sided adhesive tape was used to completely seal the chamber perimeter, thereby enhancing the reliability of our flux measurements.

As an alternative approach to the algorithm described in this section, the conventional correction algorithm employed for closed-chamber water vapor flux measurements is also presented in S5. Both methodologies yield comparable water vapor flux values. However, the algorithm in this section seems to have better agreement with the true values than the conventional algorithm for our device. Therefore, for the results presented in this paper, we use the method and correction factor presented in Figure 4, unless otherwise noted.

### Water Vapor Flux Measurements on Human Subjects with WASP

**Figure 5:**
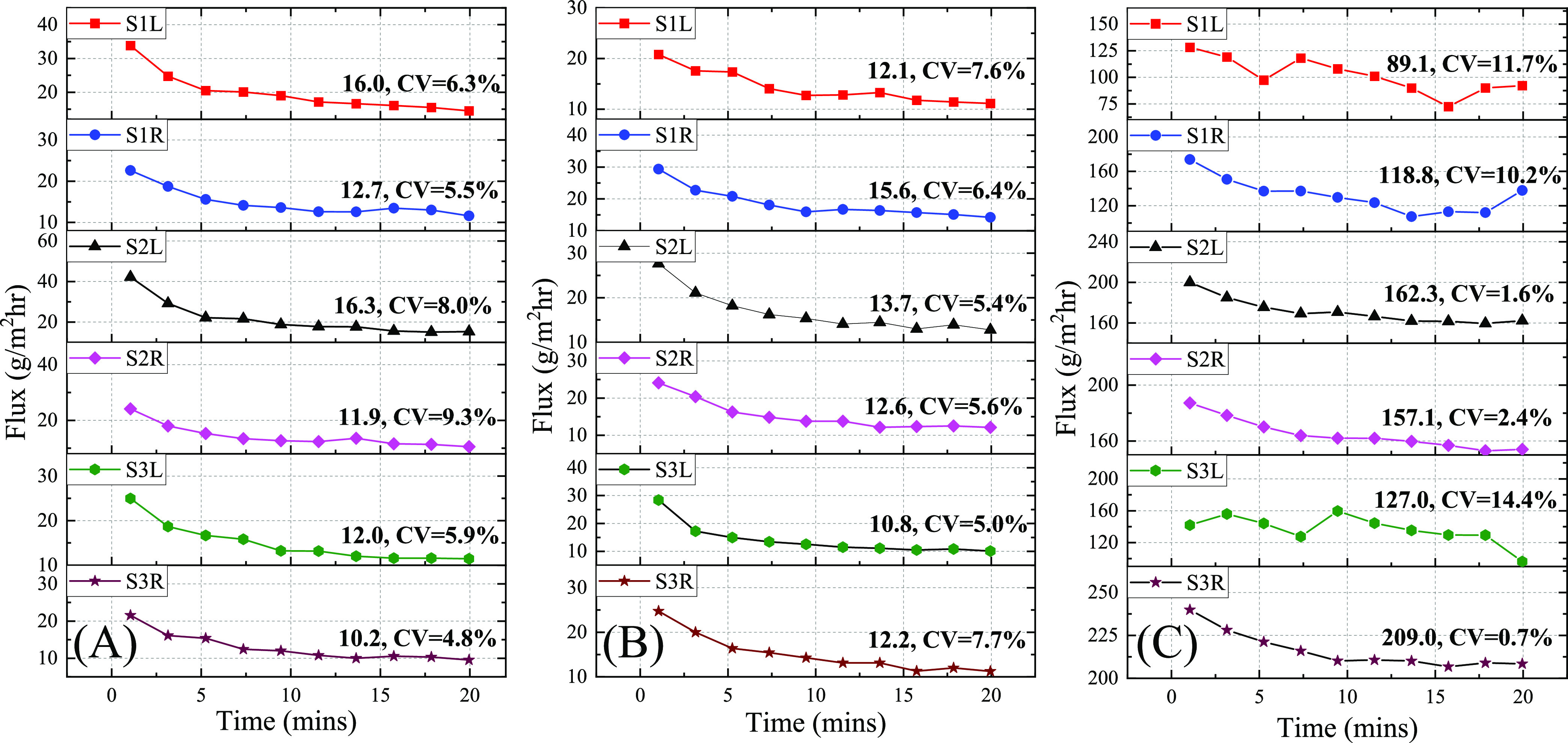
Water vapor flux measurement using WASP at three different locations: (A) upper arm, (B) forearm, and (C) palm on three human subjects. After the WASP was mounted to a body location, a waiting time of 20 min was given to allow thermal equilibrium to be established between the skin and the chamber (and the thermocouple). Then, the chamber was flushed for 60 s, followed by 60 s of RH-T reading. The entire cycle took 2 min. Ten cycles were repeated without interruption. The average flux value and the corresponding CV for the last five data points (last 10 min) are provided by each curve. S1L: Subject-1 left-hand; S1R: Subject-1 right-hand; S2L: Subject-2 left-hand; S2R: Subject-2 right-hand; S3L: Subject-3 left-hand; S3R: Subject-3 right-hand.

**Figure 6:**
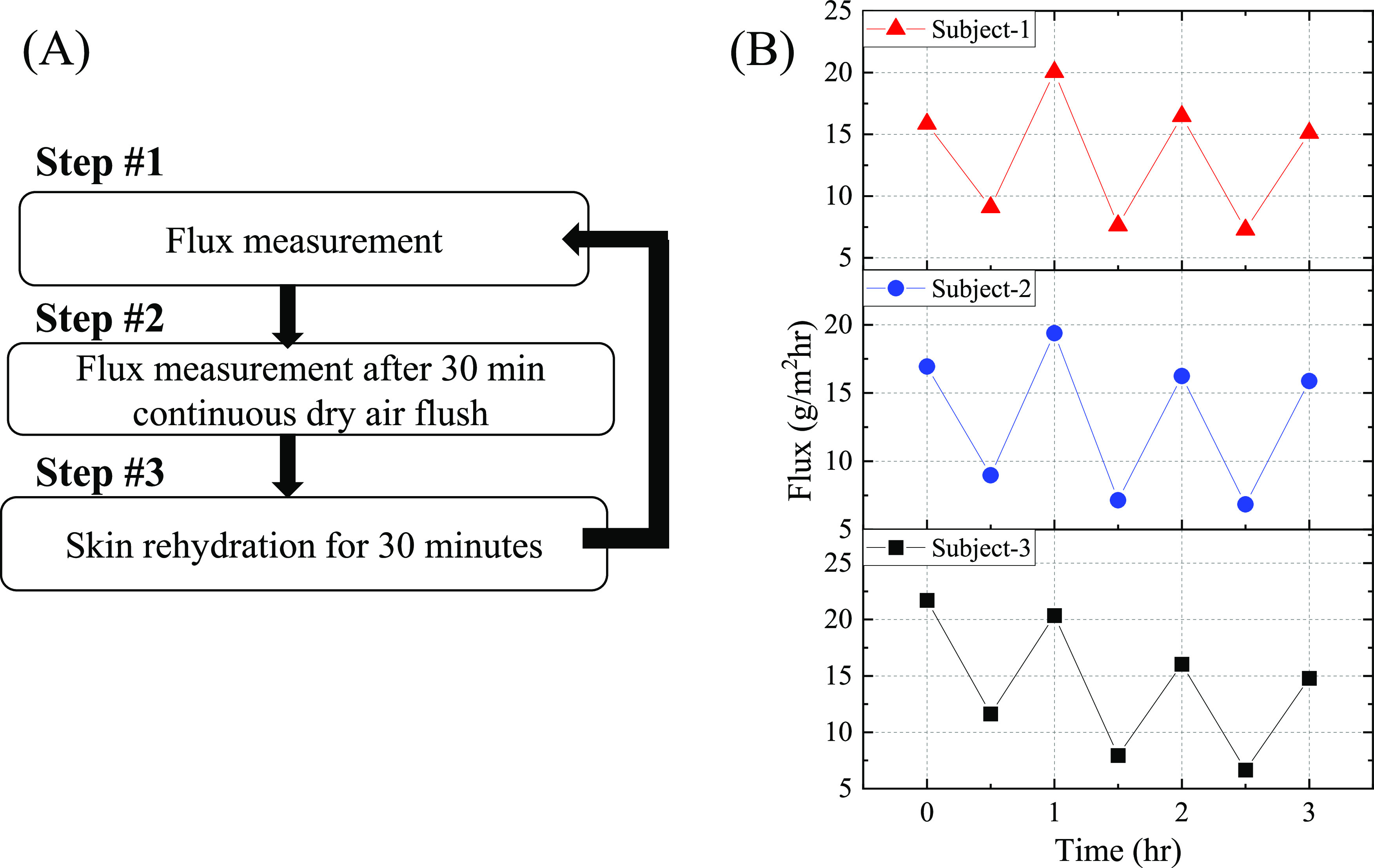
Skin hydration tracking performed at the left upper arm of three human subjects (lab members) using the WASP. (A) Experiment flow. Note that there is no waiting time. The flux measurement starts immediately after the WASP is mounted onto the skin. (B) Skin dehydration–rehydration cycles recorded using the WASP. The average flux for the three subjects at the hydration state (*i.e*., time = 0, 1, 2, and 3 h) was 16.9, 17.1, and 18.2 g/m^2^h. The average flux at the dehydration state (*i.e*., time = 0.5, 1.5, 2.5, and 3.5 h) was 8.0, 7.7, and 8.7 g/m^2^h. For comparison, TEWL of 10.1, 10.9, and 9.2 g/m^2^h were obtained with AquaFlux at the same location for the three subjects, respectively, before starting the measurement with the WASP.

Now we applied the WASP to measure water flux on six different body locations of three human subjects (lab members). Left/right upper arm (Figure 5(A)), left/right forearm (Figure 5(B)), and left/right palm (Figure 5(C)). The subjects were asked to sit in a resting position in a room of ∼23 °C. After the WASP was placed on a body location and sealed with a double-sided adhesive film, a waiting time of 20 min was given to allow thermal equilibrium to be established between the skin and the TEWL chamber (and the thermocouple). According to Figure S10, the thermocouple reading increased from room temperature to 28–32 °C, since the heat from the skin warms up the TEWL chamber and the skin. The final temperature varies slightly depending on subjects and body locations, indicative of different heat-generation rates. Note that here we used the initial 20 min of waiting time to examine how the chamber interior environment (and the skin local environment) changes when the WASP is mounted onto the skin. In practice, the waiting time can be eliminated and the flux measurement can start immediately after the WASP is mounted (see, for example, Figure 6).

After the initial waiting time, the flux measurement started. Each flux measurement cycle took 2 min, including 60 s of dry air flush at a rate of ∼90 mL/min through internal air circulation and 60 s of RH-T sensor reading. Ten cycles were repeated without interruption. As seen in Figure 5, all of the data show that the vapor flux from the skin decreases progressively during the first 5 or 6 measurements and then gradually levels off, which suggests that the water in the skin (stratum corneum layer) was gradually depleted. It is known that the water vapor flux coming out of the skin has contributions from both insensible sweating (*i.e*., TEWL) and sensible sweating (or thermal sweating) due to activated sweat gland secretion.^47^ Therefore, the initial water vapor flux values measured in Figure 5 contain contributions from both sensible and insensible sweating. Sensible sweating is inevitable when the ambient temperature surrounding the skin region under test is 28–32 °C inside the chamber.^48^ The last few flux values, when the water content in the stratum corneum layer has been significantly depleted, result mainly from the sensible sweating, which serves as the background. We further notice that the background flux for the palm is much higher than for the upper arm and forearm, since the density of sweat glands in the palm (∼520 glands/cm^2^) is higher than the upper arm (∼90 glands/cm^2^) and forearm (∼100 glands/cm^2^).^47^ Finally, the WASP exhibits good repeatability as seen in the last five flux measurements in Figure 5 that have a CV of <10% in most cases, which is due to the closed-chamber design and identical initial dry conditions inside the chamber and on the skin surface for each measurement.

The actual TEWL due to insensible sweating can be calculated by subtracting the background from the initial water flux reading (*i.e*., the flux value at t = 2 min–the averaged value of the last five readings), which is listed in Table S4. For comparison, parallel experiments were conducted using the AquaFlux device equipped with the reduced orifice cap on the same subjects and same body locations ∼3 min after the WASP measurement (see Figure S11). At this time, the skin ambient temperature for AquaFlux measurements was ∼23 °C (room temperature), which is within the AquaFlux’ specified operation temperature range of 18–28 °C, and thus sensible sweating was significantly reduced.^48^ The corresponding averaged values are listed in Table S4. In general, for upper arm and forearm measurements, the TEWL obtained by the WASP and AquaFlux match well.

For further comparison, we performed another set of experiments in which we did the flux measurement right after we mounted our device to the skin without any waiting time. In this case, the local skin was kept at room temperature (∼23 °C) at which the sensible sweating should be significantly suppressed. Additionally, we used N_2_ from a tank instead of using internal circulating air to avoid any potential heating effect from the internal circulating air and the pump. Our device measurement and AquaFlux measurement were performed alternately six times for comparison (see the procedures in Figure S12(A)). For both our device and AquaFlux measurement, the skin temperature remained nearly the same (Figure S12(B)). Figure S12(C,D) shows the flux obtained by our device and AquaFlux. As compared to the flux of 20.8 g/m^2^h measured at a higher temperature (28.2 °C) in Figure 5(B) (for S1L) using our device, which was taken at the very beginning of the experiment before any stratum corneum water depletion, the flux measured here, 7.8 g/m^2^h, is much lower due to the suppression of the sensible sweating at a lower temperature (24–25 °C). Furthermore, the flux measured by our device in Figure S12(C) matches the AquaFlux result (9.5 g/m^2^h, Figure S12(D)) well. It also matches the TEWL value for the Subject-1 left forearm in Table S4 (8.7 g/m^2^h) well. In Figure S13, another control experiment was conducted to show that the flux measurement with our device does not affect the AquaFlux measurement, and a lower skin temperature does reduce the overall flux value (due to sensible sweating).

### Skin Rehydration Tracking

By harnessing the adaptable feature of flexible-timed dry air flushes within the WASP, it also becomes possible to investigate the dynamics of stratum corneum (skin) rehydration. The incorporation of this functionality within the WASP holds significant promise for enhancing therapeutic studies involving various topical skin creams and wound healing.

To exemplify this functionality of WASP, we subjected the upper arm skin to a controlled dehydration interval of 30 min, followed by a subsequent rehydration period of 30 min. The water vapor flux values were also measured before and after dehydration. This process is illustrated in Figure 6(A). Figure 6(B) shows the water vapor flux values on the left upper arm of three human subjects (lab members) during dehydration–rehydration cycles. It can be seen clearly that the water vapor flux decreases after each 30 min of dry air purge (dehydration), suggesting the depletion of the water under the skin (in the stratum corneum layer). The remaining background water vapor flux may be attributed to sensible sweating, as discussed previously. After 30 min of rehydration, the water vapor flux almost goes back to the original value at *t* = 0 h for two subjects. For Subject-3, the water vapor flux is unable to recover fully and shows a slow decreasing trend after each dehydration cycle, suggesting a slower rehydration process than Subjects 1 and 2. The actual TEWL of 8.9, 9.4, and 9.5 g/m^2^h can be estimated by subtracting the averaged valley flux values from the averaged peak flux values for Subjects 1, 2, and 3, respectively. For comparison, the TEWL values of 10.1, 10.9, and 9.2 g/m^2^h on the same locations for Subjects 1–3 were obtained using AquaFlux before starting the measurements with the WASP. Again, for AquaFlux measurements, the skin ambient temperature was ∼23 °C at which the sensible sweating is suppressed.^48^

## Conclusions

We developed a wearable device (WASP) and the corresponding algorithm for monitoring water vapor flux emitted from skin. The unique design of the WASP avoids interference from the environment (such as ambient temperature/humidity and air flow) and ensures identical initial humidity conditions for the chamber and skin surface, all of which result in consistent results. Furthermore, the device can measure and distinguish sensible sweating and insensible sweating rates (TEWL). More importantly, TEWL measurement can be performed even when the ambient temperature is higher than 28 °C, when the existing commercial TEWL device fail to function, as at temperature above 28 °C, the sweat glands are activated, leading to strong sensible sweating that may affect the TEWL measurements. Finally, the device not only enables continuous sweat rate monitoring but also offers the ability to analyze dehydration–rehydration cycles of the skin, which is difficult for the existing TEWL devices to perform.

Further work will include the investigation of the effect of long-term (a few hours) wearing of the WASP such as skin occlusion.^49^ The occlusion for the skin directly under the WASP may be alleviated since we constantly flush the TEWL chamber. However, in the current practice, there is a large area of skin surrounding the WASP that is covered with a double-sided tape. Whether this occlusion in the surrounding skin will occur and whether occlusion will affect our TEWL measurement are yet to be answered. As a precautious step, in future measurements, we can explore the possibility of using the double-sided tape that is cloth- or water-absorbent-based to mitigate the potential occlusion issue and improve user experience during prolonged measurements.

From the device development perspective, hot or cold air (through an onboard heater and/or thermoelectric cooler) may be added to increase the WASP ability to modulate the skin sweating rate (at the expense of volume, weight, and especially, power consumption). The TEWL chambers of different opening sizes and heights will be developed and tested (for pediatric applications and for high flux measurements).

The WASP and the related method open many opportunities for clinical use for early detection of health conditions such as food allergies and various common skin disorders, and nonclinical use such as sensible sweat rate monitoring in sports, dehydration–rehydration measurement for cosmetic industries, as well as research use, such as temporal sweat pattern changes and skin behavior under various conditions, to help gain better understanding of skin dynamics. For example, numerous atopic skin conditions lack well-defined absolute ranges for assessing the severity of the condition. Instead, monitoring the progressive TEWL values and skin dynamic changes can provide critical insights for a more refined diagnostic approach.^6^ In such scenarios, the WASP will become an invaluable tool.

## Supporting Information Available

Detailed description of the approximations used in mathematical modeling; description and photographs of the experimental setup utilized for the TEWL module; characterization of the sensors used in the TEWL module and WASP; and supporting raw sensor response data plots for the tests performed on human subjects (PDF)
