# Supplementary material for: WASP: Wearable Analytical Skin Probe for Dynamic Monitoring of Transepidermal Water Loss
Source: ACS Sens. 2023 Nov 13;8(11):4407–16. doi: 10.1021/acssensors.3c01936 (PMC10683758; doi:10.1021/acssensors.3c01936)
Supplement: Supplementary file 1 — se3c01936_si_001.pdf [file se3c01936_si_001.pdf]

Supplementary Information

WASP: Wearable Analytical Skin Probe for Dynamic Monitoring of Transepidermal Water Loss

Anjali Devi Sivakumar[1,2,3,4], Ruchi Sharma[1,3,4], Chandrakalavathi Thota[1,3,4], Ding Ding[2], and Xudong Fan[1,3,4,*]

[1]Department of Biomedical Engineering,
University of Michigan, Ann Arbor, MI 48109, USA

[2]Department of Electrical Engineering and Computer Science
University of Michigan, Ann Arbor, MI 48109, USA

[3]Center for Wireless Integrated MicroSensing and Systems (WIMS[2]),
University of Michigan, Ann Arbor, MI 48109, USA

[4]Max Harry Weil Institute for Critical Care Research and Innovation
University of Michigan, Ann Arbor, MI 48109, USA

*: Corresponding author: xsfan@umich.edu

Table S1. Comparison among different commercial hygrometer-based TEWL measurement technologies and WASP.

Technology	Working Principle	Advantages	Disadvantages	Example Systems
**Open chamber**<br>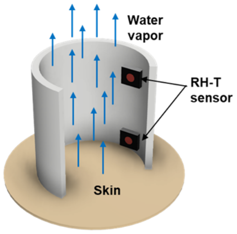 	Steady state diffusion	-Can be used for continuous monitoring.<br>-Less power consumption.	-Measurement errors due to environmental variations like airflow, ambient humidity, and temperature.<br>-Bulky and not wearable.	-DermaLab (Cortex Technology, Hadsund, Denmark)[1]<br>-Tewameter Hex (Courage+Khazaka Electronic GmbH)[2]
**Closed unventilated chamber**<br>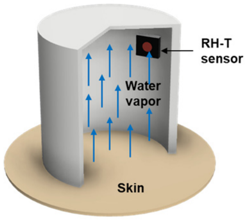 	Transient diffusion	-Measurement not affected by environmental variations.<br>-Less power consumption.	-Measurements affected by initial humidity inside the chamber and moisture on the skin surface.<br>-Cannot be used for continuous monitoring.	VapoMeter (Delfin Technology, Kuopio, Finland)[3]
**Closed condenser-based chamber**<br>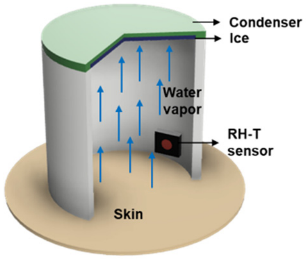 	Steady state diffusion	-Can be used for continuous monitoring but relatively shorter time than an open system because of the need for removing ice condensed in the chamber.<br>-Measurement not affected by environmental variations.	-Relatively more power consumption because of the condenser.<br>-Temperature in the chamber is low, which may cause discomfort to subjects and change the skin's inherent properties.<br>-Bulky and not wearable.	AquaFlux AF200 (Biox Systems Ltd., London, UK)[4]
**Closed ventilated chamber**<br>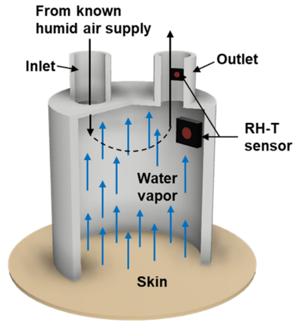 	-Steady state diffusion<br>-Humidity difference between inlet and outlet	-Can be used for continuous TEWL monitoring<br>-Measurement not affected by environmental variations.	-Bulky and not wearable since it needs extra air pumps and pneumatic systems for operation.	Q-sweat system (WR Medical Electronics Co., Maplewood, MN, USA)[5]
**Closed purge and analysis chamber**<br>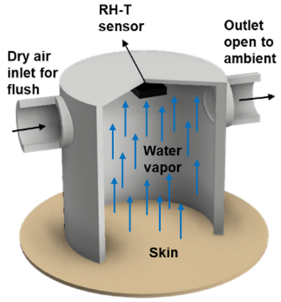 	Transient diffusion	-Wearable<br>-Can be used for continuous water vapor flux monitoring (both sensible and insensible sweating)<br>-Consumes less power compared to the condenser-based system.<br>-No ambient variation effects (such as airflow).<br>-Highly controlled environment near the measurement site – reproducible results and constant starting baseline for all the measurements.<br>-No effect on the inherent properties of the skin.<br>-Can analyze skin dehydration and rehydration.	-Filters need to be replaced periodically.	Wearable Analytical Skin Probe (WASP) – our device

Table S2. Comparison among different wearable sweat rate detection devices.

Technology	Working Principle	Advantages	Disadvantages	Reference
**Open chamber**	- Hygrometer-based device<br>-Steady state diffusion	-Can be used for continuous monitoring.<br>-Less power consumption.<br>-Can be used for insensible sweat loss (TEWL) measurements.	-Measurement errors due to environmental variations like airflow, ambient humidity, and temperature.	[6, 7]
**Closed quasi-ventilated chamber**	-Hygrometer-based device<br>-Transient diffusion	-Measurement not affected by environmental variations.<br>-Less power consumption.<br>-Can be used for insensible sweat loss (TEWL) measurements.<br>-Can be used for semi-continuous monitoring with the help of additional actuators.	-Measurements affected by initial humidity inside the chamber and moisture on the skin surface.	[8]
**Closed condenser chamber**	-Hygrometer based device<br>-Steady state diffusion	-Measurement not affected by environmental variations.<br>- Can be used for insensible sweat loss (TEWL) measurements.<br>-Less power consumption compared to the commercial closed condenser chamber device (AquaFlux, Biox systems) with the help of passive condenser like desiccants.	-Accuracy and sensitivity of this device is greatly affected by water vapor accumulation in the desiccant over time, which is similar to the problem of ice accumulation in the commercial condenser-based chamber<br>-Desiccant needs to be replaced periodically.	[9, 10]
**Absorbent material based sweat devices**	-*Absorbent materials:* filter paper, hydrogel, sponge and textile<br>-*Output signal:* electrical, colorimetric	-Low cost.<br>-Easy to scale up.	-Not reusable.<br>-Cannot be used for measuring low sweat loss measurements like insensible water loss measurement.<br>-has poor resolution and accuracy.<br>-mostly used for sweat loss volume measurement rather than sweat loss rate measurement.<br>-measurement errors caused by the obstruction of the skin resulting from the buildup of water vapor.	[11-14]
**Microfluidic based sweat devices**	-Colorimetric principle<br>-Calorimetric principle<br>-Electrical principle	-Can be used for low insensible sweat loss (TEWL) measurements.	-Measurement errors caused by the obstruction of the skin resulting from the buildup of water vapor.<br>-Colorimetric devices: not reusable, poor resolution and accuracy<br>-Calorimetric devices: high power consumption<br>-Electrical devices: huge background noise due to electrolytes in the sweat	-Colorimetric based devices: [15, 16]<br>-Calorimetric based devices: [17]<br>-Electrical based devices: [18, 19]

Table S3. Bill of materials for a WASP device.

Components	Material	Retail price
**Casing**	Top casing – PLA (34 g)	$2.26
	Bottom rubber holder + chamber gasket – Flexible resin 80A (13.53 mL)	$2.69
**TEWL chamber**	Chamber structure - High temperature resin (11.64 mL)	$2.31
**Humidity sensor**	SHTC3 with breakout board	$6.95
**Thermocouple**	K-type thermocouple	$9.95
	MCP9600 breakout board (K-type thermocouple readout)	$15.95
**Microcontroller**	2 x Seeeduino nRF52680	2 x $15.99
**Battery**	3.7 V LiPo battery	$4.87
**Pump**	XP-P2-029 pump with smart pump control module	$457.26
**Moisture filter**	Molecular sieve 5A (0.5 g)	$1.72
	Aluminum tube (30 mm height, 7 mm diameter)	$0.35
	**Total cost**	$536.29

**Table S4**. Comparison of TEWL between WASP and AquaFlux						
**Upper arm**						
	**S1L**	**S1R**	**S2L**	**S2R**	**S3L**	**S3R**
**WASP**	17.8	9.9	15.9	12.2	13.0	11.4
**AquaFlux**	14.9	13.0	12.5	18.0	9.5	9.2
**Forearm**						
	**S1L**	**S1R**	**S2L**	**S2R**	**S3L**	**S3R**
**WASP**	8.7	13.8	14.0	11.4	17.6	12.5
**AquaFlux**	13.0	18.6	11.7	14.1	11.3	14.8
**Palm**						
	**S1L**	**S1R**	**S2L**	**S2R**	**S3L**	**S3R**
**WASP**	39.1	55.1	37.8	30.1	15.0	30.9
**AquaFlux**	32.4	44.0	48.3	52.6	44.8	44.8
Unit: g/m^2hr						

Table S4. Comparison of the TEWL values obtained from WASP and AquaFlux. The WASP TEWL values are calculated by subtracting the background from the initial water vapor flux value (*i.e.*, the flux value at t=2 min – the averaged flux value of the last 5 readings) in Figure 5 in the main text. AquaFlux TEWL values are calculated by averaging the measurements in Figure S11, which were conducted ~3 minutes after the WASP measurements for approximately 10 minutes.

S1. Mathematical Modeling

S1.1. Validating applicability of the open chamber model to a closed chamber setup

To validate the initial dynamics agreement between open and closed chamber systems, simulations were conducted using COMSOL Multiphysics 6.0.2. These simulations modeled the behavior of water vapor in both systems, with a glass beaker containing water as the vapor source. The Moisture Transport in Air module was employed to simulate water vapor diffusion dynamics within the chamber. Additionally, the Heat Transfer in Moist Air module was used to simulate water evaporation dynamics at varying temperatures to generate different water fluxes.

The outcomes of these simulations are depicted in Fig. S1. Notably, near time zero (~0.1 s), the peak flux values are the same between the open and closed systems. However, as time progresses beyond zero, a distinct contrast arises. In the open system, the flux value stabilizes at a non-zero steady-state value, while the closed system's flux value gradually approaches zero, which is due to the establishment of an equilibrium between evaporation and condensation within the chamber. It is apparent that the chamber volume may affect the establishment of the equilibrium, as shown in Fig. S1(D). Nevertheless, the peak flux value around time zero remains unchanged regardless of the volume of the closed chamber and is the same as that in the open chamber system whose volume can be treated as infinite. The independence of the peak flux value with respect to the chamber volume is due to the fact that it takes time for equilibrium of water vapor to be established within the chamber. As long as the chamber height is reasonably large (which is applicable to our chamber that had a height of 4.75 mm), the initial water vapor flux from the water/air interface does not have time to reach the chamber ceiling, and therefore, sees the chamber to have an infinite height. In our algorithm we use the peak value in the flux temporal response curve as the measured water vapor flux. Therefore, Eq. (2) in the main text used for an open chamber system is applicable to a closed chamber system.

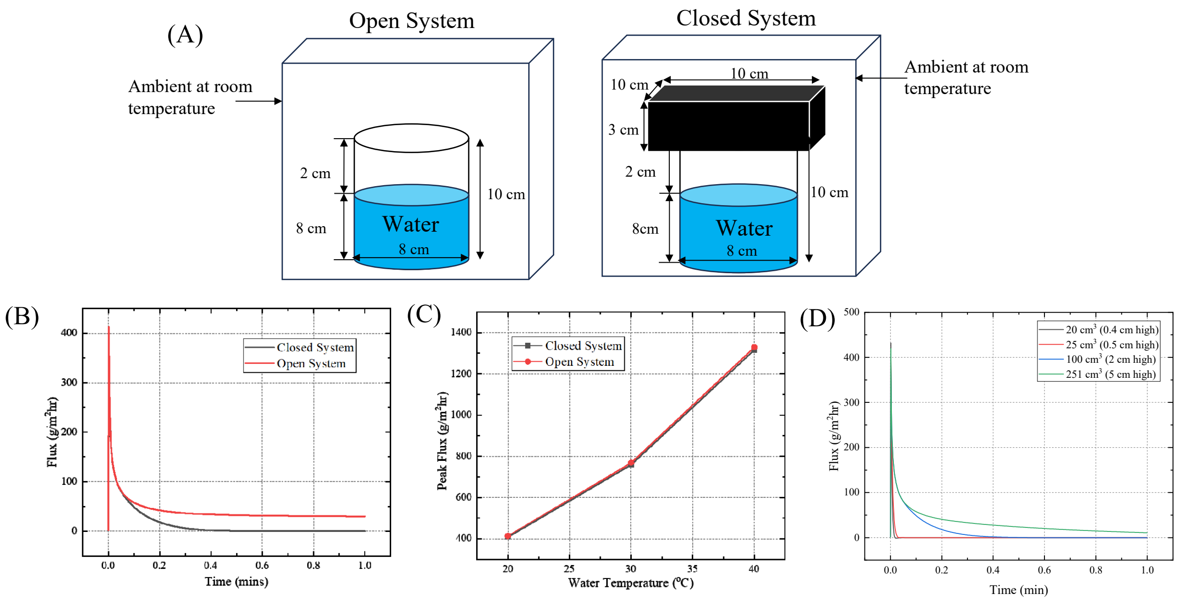

Figure S1. COMSOL simulation for comparison of the open and closed system. (A) COMSOL model setup. (B) Flux curves obtained at 0.4 mm above the 20 ºC water surface. (C) Comparison of peak flux values recorded with different water temperatures for both open and closed systems at 0.4 mm above the water surface. (D) Comparison of flux temporal responses in the closed chamber with different chamber heights (volumes) at 4 mm, 5 mm, 20 mm, and 50 mm. All the peak flux values are the same as that obtained with an open system in (A) under otherwise the same conditions.

S1.2. Determination of the spatial point for C_2

As shown in Eq. (3) and Fig. 1(B) in the main text, we need two water vapor concentrations (C_1 and C_2) at two different spatial points close to the skin surface to calculate the flux. C_1 can be chosen as the skin surface (*i.e.*, y=0 mm). Below we conducted simulations using MATLAB Version 2022b (MathWorks) to determine the spatial point for C_2.

In this simulation, water (at temperature of 20 ºC) was used as a constant and non-depleting water vapor source in the flux model simulation as depicted the Fig. S2(A). The results in Fig. S2(B) illustrate the simulation outcomes of the water vapor concentration profile at various spatial points along the height of the chamber as time progresses. It is evident from this plot that the water vapor concentration gradient along the chamber's height becomes linear over time. However, at times very close to zero and at spatial points near the boundary, *i.e.,* the surface of the water vapor source, y=0, the water vapor concentration profiles exhibit nonlinear behavior due to the omission of solute particle inertia in the Fick's model at the boundary. The Fick's diffusion model fails to address the ballistic motion near boundaries, leading to an infinite water vapor concentration gradient[20]. Several transport mechanism models, like Cattaneo's diffusion[20] and telegraphic transport models[21], were developed to tackle the infinite gradient issue at the boundaries. These transport models assume that particles propagate with finite velocities at the boundaries for short times whereas they become diffusive at longer times when random collisions have been able to thermalize the particle's motion[20, 21]. However, Cattaneo's diffusion and telegraphic models are analytically complex. Therefore, we adopted a simpler alternative approach in which the spatial point for C_2 was placed at a distance far enough from the water source so that the Fick model becomes valid. Based on our modeling, it is found that at any spatial point above 2 mm (*i.e.*, y >= 2mm), the flux calculated using our dynamic mathematical model and that obtained from the steady-state mathematical model based on the MATLAB simulations match very well (<1% difference). In our algorithm, we used y=2 mm for C_2.

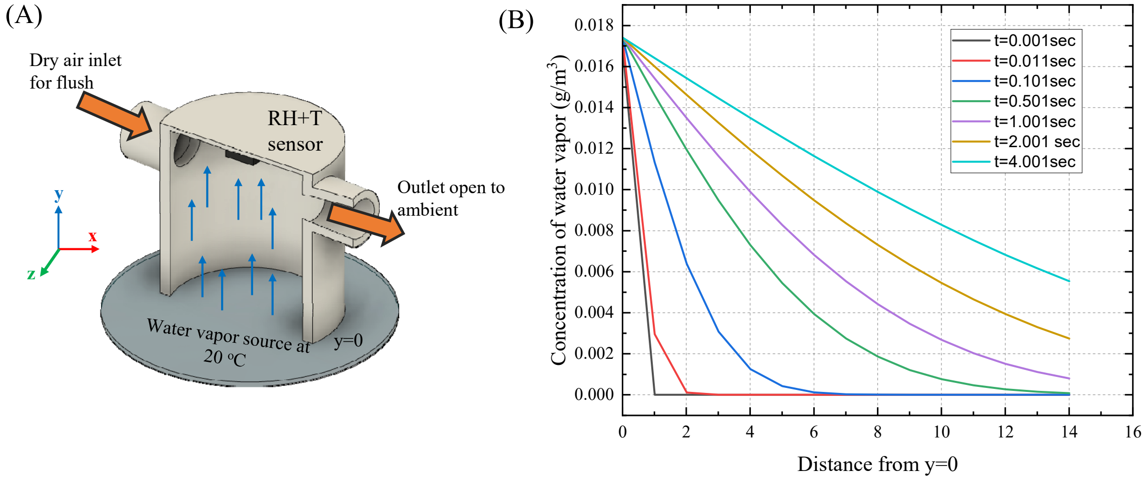

Figure S2. MATLAB simulation of Fick's second law applied to our flux model with a water vapor source at temperature 20 ºC to illustrate the ballistic motion of the particles near the boundary of y=0. (A) Flux model used for simulation. (B) Water vapor concentration profiles along the height of the chamber at different times.

S1.3. Relative humidity sensor delay

Here, a simple first-order delay model was added to RH sensor readings (see Fig. S3(A)) in the MATLAB simulation of our mathematical model performed in S1.1 to illustrate the effect of RH sensor delay on the flux measurements. It can be seen from Fig. S3(B) that the RH sensor delay significantly affects the flux measurements' accuracy (in both the peak value and the time to reach the peak value). Hence, a correction factor should be employed in our flux estimation protocol to account for the delay in the RH sensor readings.

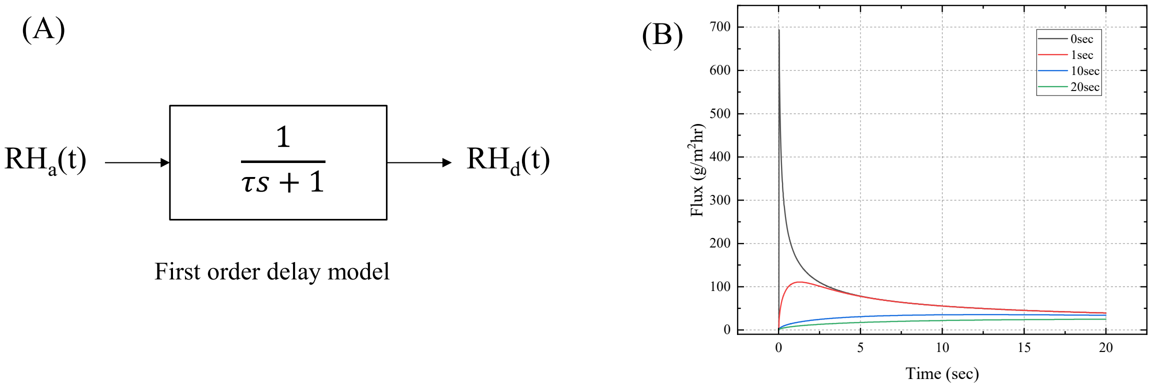

Figure S3. MATLAB simulation of Fick's second law applied to our flux model with a constant water vapor source illustrates the effect of RH sensor delay on the accuracy of the flux measurements. (A) Simple first order delay model in frequency domain (s-domain) for the RH sensor. τ is the time constant. (B) Temporal flux obtained with an RH sensor with different response times/delays.

S2. Materials and Methods

To characterize the proposed water vapor flux measurement device, we first constructed a TEWL module that had a 3D-printed chamber with dimensions of 4.5 mm (width) × 8 mm (length) × 4.75 mm (height) using High-Temperature Resin (P/N: RS-F2-HTAM-02, Formlabs) (see Fig. S4(A)). A capacitive polymer-based RH sensor (SHTC3, Sensirion) and the corresponding breakout board (Adafruit) were used to monitor the RH transients. The sensor was placed on top of the chamber ceiling. The bare electronics exposed on the sensor board were protected with a waterproof plastic wrap. Further, to ensure a tight seal between the board and the chamber, a flexible 3D-printed gasket using Flexible 80A resin (P/N: RS-F2-FL80-01, Formlabs) was sandwiched between the sensor board and the chamber as shown in Fig. S4(B). Since the integrated temperature sensor in SHTC3 was observed to provide inaccurate temperature readings in the presence of a non-uniform heat source, for instance, skin, an external K-Type thermocouple (P/N: 270, Adafruit) was installed near the SHTC3 to measure temperature transients more accurately inside the chamber (near the RH sensor). The thermocouple was capped with a single layer of Kapton tape (substrate thickness – 0.025 mm and adhesive thickness – 0.05 mm; P/N: EL-CP-022, Elegoo) to protect the thermocouple from moisture accumulated in the chamber (Fig. S4(C)). The addition of Kapton tape on the thermocouple was shown to have no impact on the thermocouple's final temperature reading. The temperature was recorded with a breakout board with MCP9600 (P/N: 4101, Adafruit). The RH and temperature transient data from the sensors was streamed to a laptop in real time with Seeed Studio nRF52840 Sense (Microcontroller Unit) using LabVIEW. Further, the time-controlled dry air or N_2 flushes were provided to the chamber from an external tank with the help of two electromechanical valves (P/N: LFVA1220210H, LEE Company).

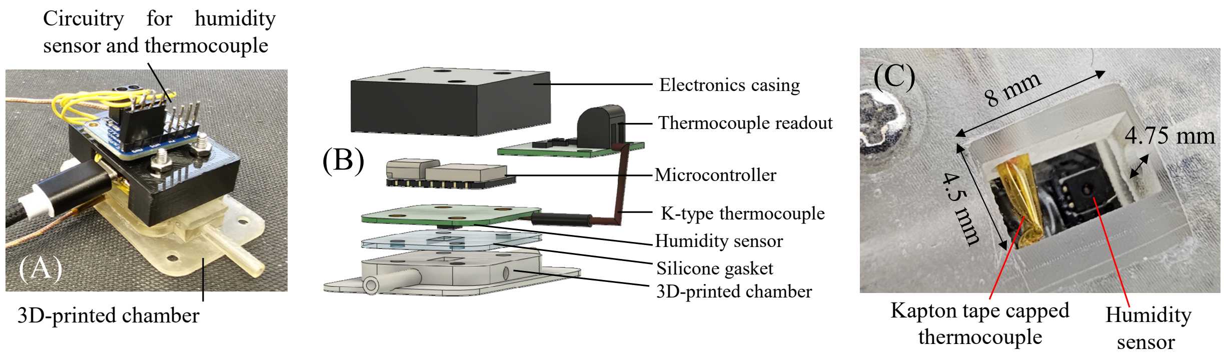

Figure S4. TEWL module design. The RH sensor and the thermocouple were placed on the top of the chamber. The thermocouple was capped with a Kapton tape to protect it from moisture. (A)-(C) Top, exploded, and bottom of view of the TEWL module. The chamber had an opening 8 mm x 4.5 mm to the skin. The chamber's inner height was 4.75 mm.

S3. RH Sensor Characterization

S3.1. RH sensor repeatability

As shown in Fig. S5(A), a wireless mug with integrated heater (Vsitoo, Amazon) filled with water and covered by a semi-permeable membrane (304 Stainless Steel 150 Mesh, Uxcell) was used to provide a constant and non-depleting source of water vapor. This setup was employed to evaluate the repeatability of the RH sensor's response. The temperature of the water in the mug was set to 43 °C. To conduct this experiment, the TEWL chamber setup along with the dry air supply tank described in the "Materials and Methods" section in the main text was utilized.

The chamber, with its opening facing the water vapor source, was positioned on the semi-permeable membrane, as illustrated in Fig. S5(A). Before initiating the experiment, a 20-minute period was allotted for the chamber to reach thermal equilibrium with the water vapor source. Following this, 60-s dry air flush was applied to the chamber before starting the actual water vapor flux experiment. Throughout the experiment, twelve consecutive RH sensor curves were recorded, each spanning 60 seconds, with intermediate 60-s intervals of dry air flush to eliminate any accumulated water vapor within the chamber. Fig. S5(B) presents the RH sensor response curves, demonstrating a high level of repeatability in RH sensor response. For example, CV is 2.52% and 1.64% for t = 10 s and 20 s, respectively.

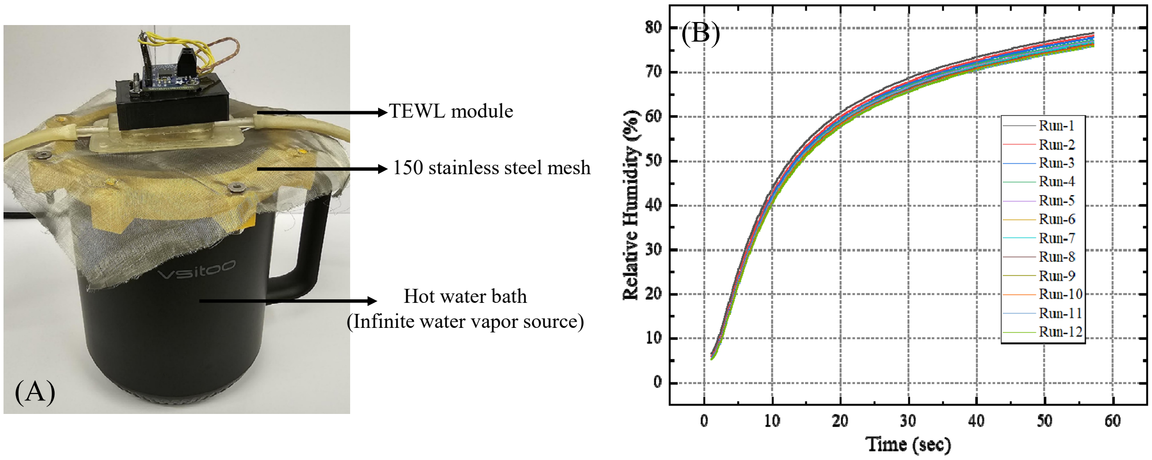

Figure S5. Repeatability test for the onboard relative humidity (RH) sensor in SHTC3 in the presence of a non-depleting and constant water vapor source. (A) Experimental setup. (B) Temporal response of RH sensor for 12 consecutive measurements performed after 20 minutes of water temperature stabilization at 43 ºC. CV = 2.52% at t = 10 s. CV = 1.64% at t = 20 s.

S3.2. RH sensor response time

The response time assessment of the SHTC3's RH sensor was carried out using a conventional bubbler setup, as illustrated in Fig. S6(A). To conduct this experiment, the TEWL chamber setup described in the "Materials and Methods" section of the main text was employed. Two different sizes of TEWL chambers were utilized: 4 mm × 4.5 mm × 4.75 mm (85.5 µL – small chamber) and 4.5 mm × 8 mm × 4.75 mm (171 µL – standard chamber).

To ensure airtight conditions, the opening of the TEWL chamber was securely sealed using a 3D-printed lid and gasket. One of the outlets of the TEWL chamber was connected to the output of the bubbler setup, while the other outlet remained open to the ambient, as indicated in Fig. S6(A). Moist air with varying levels of RHs with intermediate dry air flushes was introduced into

the TEWL chamber at a rate of 300 sccm, as shown in Figs. S6(B) and (D).

In order to determine the response time of the sensor at different RH levels, the RH sensor responses are first normalized. The time at which the RH sensor response reaches 63% of the maximum RH value is noted as the approximate response time for the corresponding RH level. The response times of the RH sensor for the designated RH values are then plotted for both chamber sizes, as depicted in Figs. S6(C) and (E). It is observed that regardless of the chamber's volume, the response time of the RH sensor exhibits a nonlinear correlation with the RH value. This nonlinear behavior is likely attributed to the occurrence of water vapor condensation on the sensor's surface[22, 23], particularly at elevated RH levels. The nonlinear time constant with respect to the RH level calls for a correction factor for our device, which is discussed in the main text.

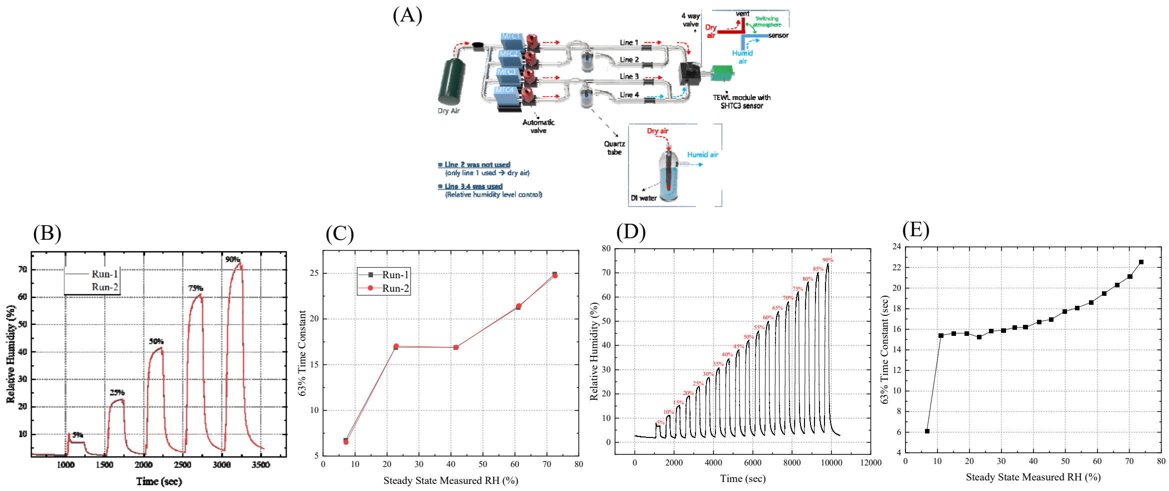

Figure S6. (A) Schematic of the bubbler test setup used to test the response time of the onboard RH sensor in SHTC3. (B) RH sensor response for two consecutive runs for a standard chamber (4.5 mm × 8 mm × 4.75 mm). (C) Response time of the RH sensor as a function of the measured relative humidity in (B). (D) RH sensor response for two consecutive runs for a small chamber (4 mm × 4.5 mm × 4.75 mm). (E) Response time of the RH sensor as a function of the measured relative humidity in (D).

S4. Temperature Sensor Characterization

S4.1. SHTC3's temperature sensor vs. Kapton-tape capped K-type thermocouple

The assessment of the SHTC3's temperature sensor performance was conducted in conjunction with a K-type thermocouple covered by a Kapton tape using both uniform and non-uniform heat sources. Both SHTC3's temperature sensor and the thermocouple were housed in the TEWL module and close to each other.

For the uniform heat source experiment, a conventional gas chromatography (GC) oven was used and the oven temperature was ramped (Fig. S7(A)). The temperature response curves of both the thermocouple and the SHTC3's temperature sensor were found to be identical under the conditions of a uniform heat source, as observed in Fig. S7(A), although it took some time for both sensors to reach thermal equilibrium.

Conversely, in scenarios of a non-uniform heat source, such as skin, a different setup was employed. The TEWL chamber, equipped with the SHTC3 sensor board and the Kapton tape capped K-type thermocouple, was affixed to the skin surface using double-sided tape (see Fig. S7(B)). The temporal response of both temperature sensors was simultaneously recorded. As shown in Fig. S7(B), the SHTC3's temperature sensor exhibits relatively longer response time and lower accuracy than the thermocouple. This disparity can be primarily attributed to the sensor configurations. The thermocouple's measuring tip was fully immersed inside the TEWL chamber, ensuring more accurate measurements. However, in the case of the SHTC3's temperature sensor, one side of the sensor board faced the TEWL chamber and the skin while the other side was exposed to the ambient environment. Consequently, the SHTC3's temperature sensor reading was strongly affected by the ambient temperature. Due to the important role of accurate temperature measurements in estimating absolute humidity within the TEWL chamber, the Kapton tape capped thermocouple was chosen as the temperature sensor over the SHTC3's temperature sensor. The reason to use a Kapton tape to cover the thermocouple measuring tip is

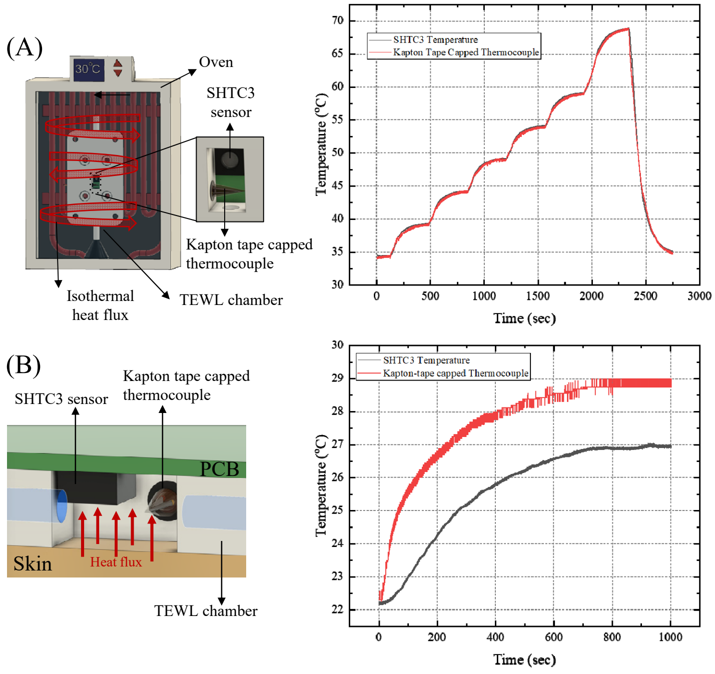

Figure S7. Comparison of the onboard temperature sensor in SHTC3 and the Kapton tape capped K-type thermocouple. (A) Uniform heat source – oven. (B) Non-uniform heat source – skin.

to avoid potential damage of the thermocouple caused by moisture since the thermocouple is frequently exposed to water vapor from skin.

S4.2. Characterization of Kapton tape capped K-type thermocouple

The impact of employing a Kapton tape cap covered on a K-type thermocouple was also investigated using a setup with a similar uniform heat source as in the previous section. This characterization experiment involved three distinct thermocouple configurations, as depicted in Fig. S8(A).

In Configuration I, a bare thermocouple was mounted to the wall of a conventional GC oven. In Configuration II, a bare thermocouple was positioned in the slot on the interior wall of a TEWL chamber. In Configuration III, a Kapton tape capped thermocouple was positioned in the slot on the same TEWL chamber in Configuration II. To evaluate these configurations, the oven temperature was ramped.

Fig. S8(B) shows that both Configuration II and Configuration III exhibited increased response time compared to Configuration I, which can primarily be attributed to the reduced thermal conductance arising from the small opening of the TEWL chamber and the Kapton tape. However, it is important to note that temperature readings across all three configurations were consistent once steady-state conditions were achieved, even with the Kapton tape capped thermocouple.

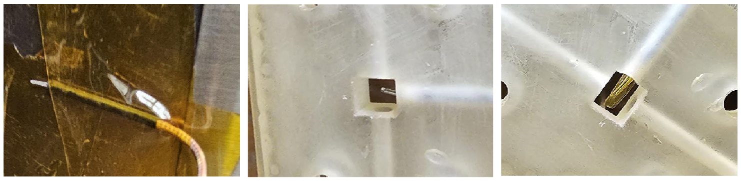

(I) Bare thermocouple

(II) Bare thermocouple inside a TEWL chamber

(III) Kapton tape capped thermocouple inside a TEWL chamber

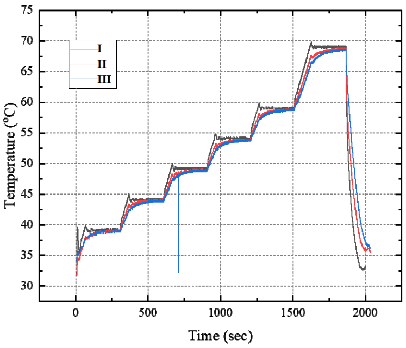

Figure S8. Characterization of accuracy and response time of a Kapton tape capped K-type thermocouple against a bare K-type thermocouple.

S5. Alternative correction algorithm

Alternatively, we can also use the conventional method to correct the water vapor flux obtained by our device. The conventional correction algorithm for closed chamber TEWL devices involves utilizing wet-cup experiments to establish a linear correlation between the actual flux value (determined through weighing scale measurement) and the slope of absolute/relative humidity derived from RH-T sensor's dynamic response. This linear correlation acts as a foundation for predicting flux values once the measurement's absolute/relative humidity slope is known[3, 8].

In this context, we present the application of this correction algorithm using the slope of the temporal response of absolute humidity obtained from our TEWL module. Fig. S9(A) shows the correction curve. The true flux value data were obtained from the weighing scale measurements in the wet-cup experiments. The absolute humidity slope is the average of the slopes calculated at three points positioned at 2 s, 4 s, and 6 s prior to the flux apex time in the TEWL curve measured by our TEWL module. It is interesting to notice that the fit curve has nearly 0 intersection due to the initial dry conditions inside the chamber and on the skin surface resulting from the purge. In contrast, the calibration curve starts to bend downwards in Fig. 5 of Ref. 8, that is, the RH slope decreases more at a low flux, indicative of the effect of the initial humidity inside the chamber and on the skin surface.

Fig. S9(B) shows the flux values for seven flux data points generated using wet-cup experiments, both with the AquaFlux device and our TEWL module (computed using the linear fit correction curve from Fig. S9(A)). It appears that both calibration methods (Figs. 5 and S9) produce similar flux measurement with our device that in general agreement with the true flux measured by the weighing scale method.

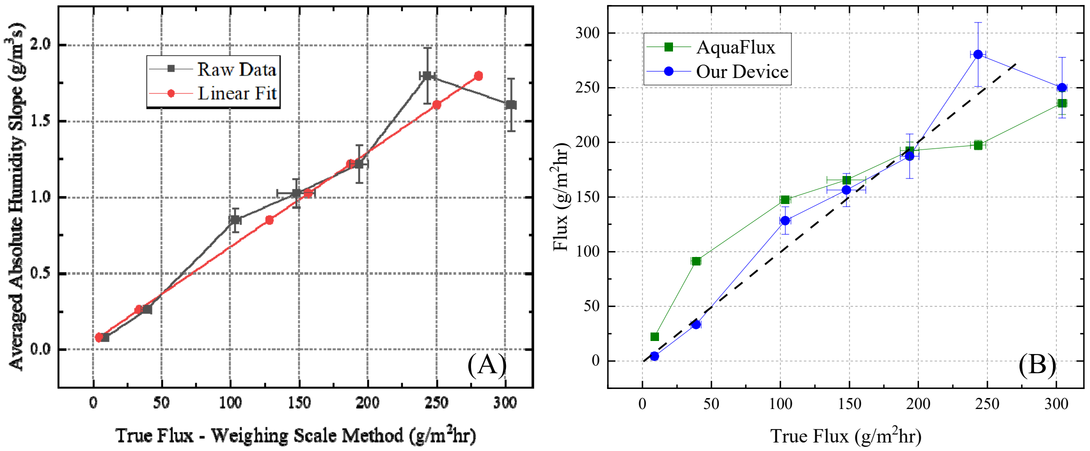

Figure S9. (A) Averaged absolute humidity slope vs. the true flux measured by a weighing scale. The red curve is the linear fit between 8 g/m^2hr and 300 g/m^2hr). Error bars are obtained from at least 10 consecutive measurements. (B) Comparison of flux measurements from three different devices – weighing scale (true flux), our device (whose flux values are corrected with the averaged absolute humidity slope in the red curve in (A), and AquaFlux whose error bars are obtained from at least five consecutive measurements. The black dashed line shows a perfect match between the measured and the true flux.

S6. WASP

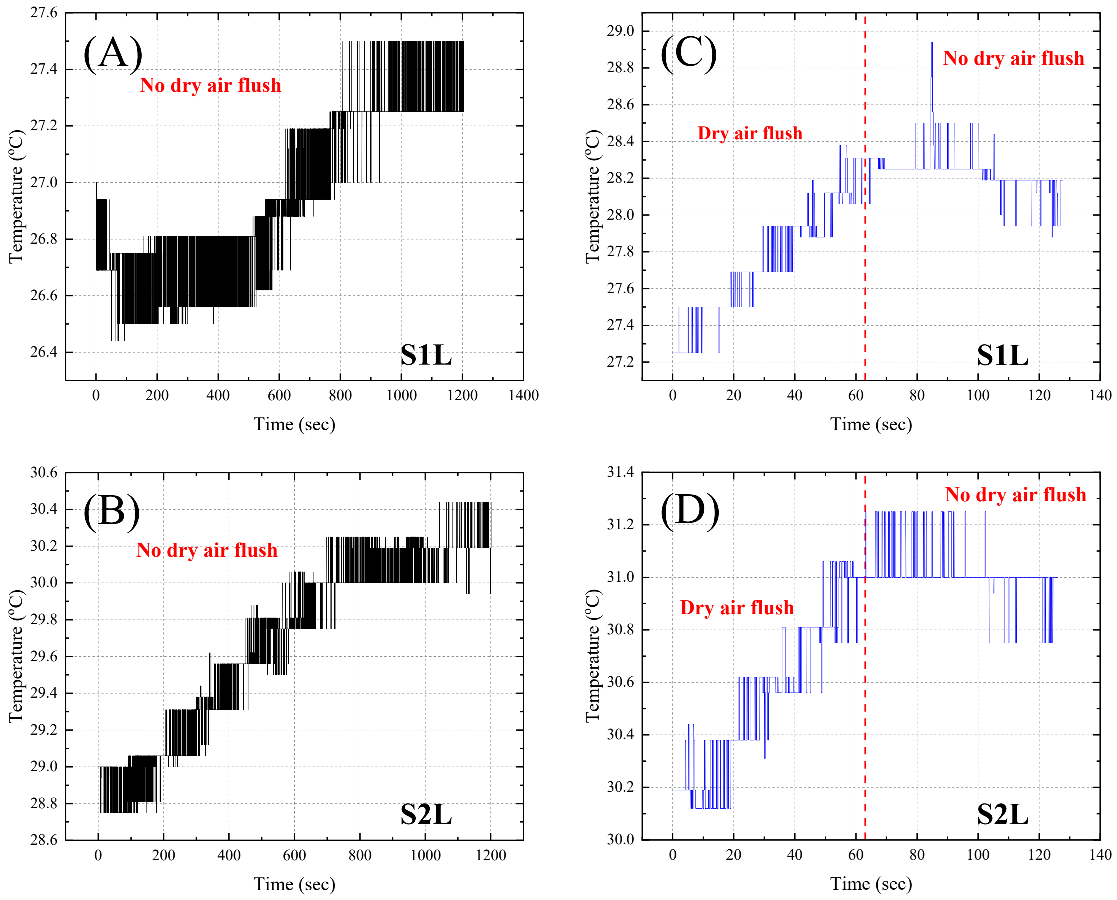

Figure S10. Exemplary temperature transients recorded inside the chamber at the forearm of two subjects (A)-(B) during the initial 20 mins skin warm-up period without dry air flush. (C)-(D) during the first water vapor flux measurement conducted immediately after the 20-minute skin warm-up phase. The entire flux measurement process comprises 60-second dry air flush, followed by 60-second flux measurement without dry air flush. S1L: Subject-1 left forearm; S2L: Subject-2 left forearm. The red dashed lines in (C) and (D) mark the time when the dry air purge stopped and the flux measurement started.

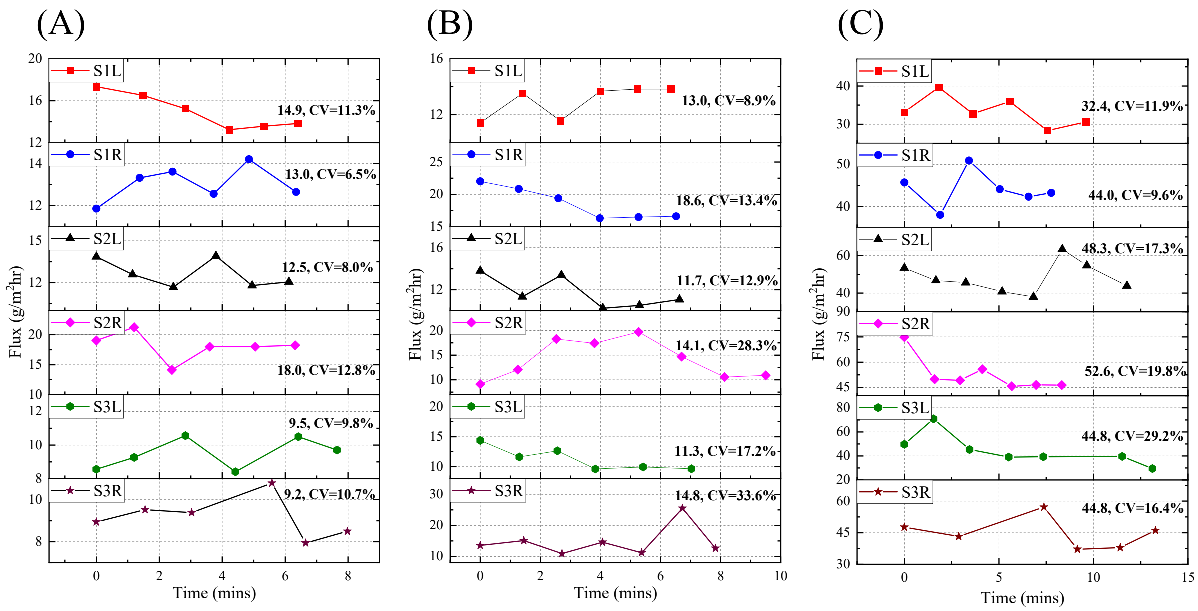

Figure S11. TEWL measurement using AquaFlux on body locations: upper arm (A), forearm (B), and palm (C) in the same three human subjects corresponding to Figure 5. The AquaFlux measurements were conducted using the reduced orifice right after the WASP measurements. After each AquaFlux measurement, the device was removed from the skin and put back on for the next measurement. The average TEWL value and the corresponding CV are provided by each curve.

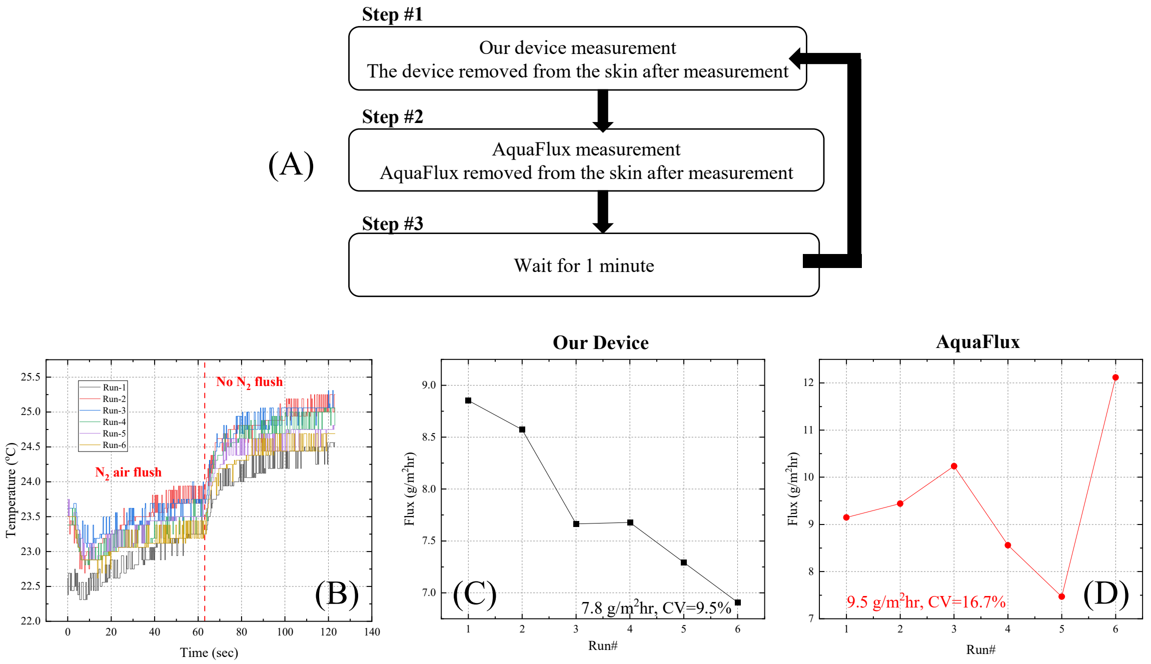

Figure S12. Comparison between our device measurement and AquaFlux measurement on left forearm of Subject-1 at the same skin temperature that is close to the room temperature. (A) Experimental procedures. Note that there was no initial waiting time. Our device measurement started right after the device was mounted onto the skin. (B) Chamber temperature (or skin temperature) recorded by the thermocouple during six measurements using our device. The overall chamber temperature was in the range of 23.5 ºC and 25 ºC. During each measurement, the chamber temperature increased by ~0.5 ºC from the beginning to ~30 s where the flux apex was reached. The red dashed line indicates the time when the purge stopped and the flux measurement started. (C) Flux measured by our device. The average flux and the corresponding CV are given by the curve. (D) Flux measured by AquaFlux equipped with the reduced orifice cap. The skin temperature for AquaFlux measurement was the room temperature (~23 ºC). The average flux and the corresponding CV are given by the curve.

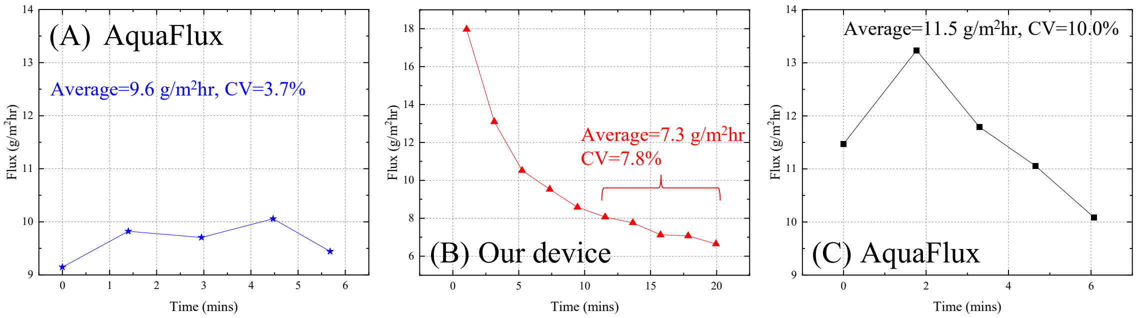

Figure S13. Another control experiment to examine whether the flux measurement with our device affects the subsequent AquaFlux measurements, and whether a lower skin temperature reduces the flux reading in our device. The experiment was conducted on Subject-1 left forearm on a different day from when the results in Figures 5 and S12 were obtained. (A) AquaFlux measurements (5 times) were first conducted. (B) Then the flux measurements (10 times) were conducted using our device and following the same protocol used in Figure 5. (C) AquaFlux measurement (5 times) was conducted ~3 minutes after the last measurement in (B) in the same manner as in (A). The averaged flux values and the corresponding CVs for AquaFlux measurements before and after our device measurement are given by the curves in (A) and (C), respectively. The averaged flux value for the last 5 measurements using our device and the corresponding CV are given in (B). The TEWL obtained by our device is the flux value at t = 2 min – 7.3 (average of the last 5 flux values) = 10.6 g/m^{2}hr, which agrees with the AquaFlux results of 9.6 g/m^{2}hr and 11.5 g/m^{2}hr before and after our device measurement. Note: immediately after the AquaFlux measurement in (C), we used our device to measure the skin flux again. This time, there was no 20-min of waiting time and the skin temperature was about 23-25 ^{o}C (similar to the skin temperature when AquaFlux measurements were conducted), at which sensible sweating was suppressed. The flux (*i.e.*, TEWL) obtained by our device is 8.8 g/m^{2}hr.

References:

(1) *DermaLab single*. Cortex Technology, https://cortex.dk/single-parameter-dermalab/ (accessed Nov 2022).

(2) *Tewameter TM Hex*. Courage+Khazaka electronic GmbH, https://www.courage-khazaka.de/en/scientific-products/tewameter-tm-hex (accessed Nov 2022).

(3) Nuutinen, J.; Alanen, E.; Autio, P.; Lahtinen, M. R.; Harvima, I.; Lahtinen, T. A closed unventilated chamber for the measurement of transepidermal water loss. *Skin Research & Technology* **2003**, *9* (2), 85-89.

(4) Imhof, R. E.; Berg, E. P.; Chilcott, R. P.; Ciortea, L. I.; Pascut, F. C.; Xiao, P. New Instrument for Measuring Water Vapor Flux Density from Arbitrary Surfaces. In *IFSCC Magazine*, 2002; Vol. 5, pp 297-301.

(5) *Q-Sweat (Quantitative Sweat Measurement System)*. WR Medical Electronics Co., 2020. https://wrmed.com/wp-content/uploads/2021/03/Q-Sweat-Instructions-for-Use-Rev_11.pdf (accessed Nov 2022).

(6) Salvo, P.; Di Francesco, F.; Costanzo, D.; Ferrari, C.; Trivella, M. G.; De Rossi, D. A Wearable Sensor for Measuring Sweat Rate. *IEEE Sensors Journal* **2010**, *10* (10), 1557-1558.

(7) Salvo, P.; Pingitore, A.; Barbini, A.; Di Francesco, F. A wearable sweat rate sensor to monitor the athletes' performance during training. *Science & Sports* **2018**, *33* (2), e51-e58.

(8) Sim, J. K.; Yoon, S.; Cho, Y. Wearable Sweat Rate Sensors for Human Thermal Comfort Monitoring. *Scientific Reports* **2018**, *8* (1), 1181.

(9) Ogai, K.; Fukuoka, M.; Kitamura, K.; Uchide, K.; Nemoto, T. A Detailed Protocol for Perspiration Monitoring Using a Novel, Small, Wireless Device. *Journal of Visualized Experiments* **2016**, (117).

(10) Gao, K.-P.; Shen, G.-C.; Zhao, N.; Jiang, C.-P.; Yang, B.; Liu, J.-Q. Wearable Multifunction Sensor for the Detection of Forehead EEG Signal and Sweat Rate on Skin Simultaneously. *IEEE Sensors Journal* **2020**, *20* (18), 10393-10404.

(11) Vaquer, A.; Barón, E.; de la Rica, R. Wearable Analytical Platform with Enzyme-Modulated Dynamic Range for the Simultaneous Colorimetric Detection of Sweat Volume and Sweat Biomarkers. *ACS Sensors* **2021**, *6* (1), 130-136.

(12) Huang, X.; Liu, Y.; Chen, K.; Shin, W.-J.; Lu, C.-J.; Kong, G.-W.; Patnaik, D.; Lee, S.-H.; Cortes, J. F.; Rogers, J. A. Stretchable, Wireless Sensors and Functional Substrates for Epidermal Characterization of Sweat. *Small* **2014**, *10* (15), 3083-3090.

(13) Yang, Y.; Xing, S.; Fang, Z.; Li, R.; Koo, H.; Pan, T. Wearable microfluidics: fabric-based digital droplet flowmetry for perspiration analysis. *Lab on a Chip* **2017**, *17* (5), 926-935.

(14) Zhao, F. J.; Bonmarin, M.; Chen, Z. C.; Larson, M.; Fay, D.; Runnoe, D.; Heikenfeld, J. Ultra-simple wearable local sweat volume monitoring patch based on swellable hydrogels. *Lab on a Chip* **2020**, *20* (1), 168-174.

(15) Koh, A.; Kang, D.; Xue, Y.; Lee, S.; Pielak, R. M.; Kim, J.; Hwang, T.; Min, S.; Banks, A.; Bastien, P.; et al. A soft, wearable microfluidic device for the capture, storage, and colorimetric sensing of sweat. *Science Translational Medicine* **2016**, *8* (366).

(16) Choi, J.; Bandodkar, A. J.; Reeder, J. T.; Ray, T. R.; Turnquist, A.; Kim, S. B.; Nyberg, N.; Hourlier-Fargette, A.; Model, J. B.; Aranyosi, A. J.; et al. Soft, Skin-Integrated Multifunctional Microfluidic Systems for Accurate Colorimetric Analysis of Sweat Biomarkers and Temperature. *ACS Sensors* **2019**, *4* (2), 379-388.

(17) Kwon, K.; Kim, J. U.; Deng, Y.; Krishnan, S. R.; Choi, J.; Jang, H.; Lee, K.; Su, C.-J.; Yoo, I.; Wu, Y.; et al. An on-skin platform for wireless monitoring of flow rate, cumulative loss and temperature of sweat in real time. *Nature Electronics* **2021**, *4* (4), 302-312.

(18) Nyein, H. Y. Y.; Bariya, M.; Tran, B.; Ahn, C. H.; Brown, B. J.; Ji, W.; Davis, N.; Javey, A. A wearable patch for continuous analysis of thermoregulatory sweat at rest. *Nature Communications* **2021**, *12* (1).

(19) Yuan, Z.; Hou, L.; Bariya, M.; Nyein, H. Y. Y.; Tai, L.; Ji, W.; Li, L.; Javey, A. A multi-modal sweat sensing patch for cross-verification of sweat rate, total ionic charge, and Na+ concentration. *Lab on a Chip* **2019**, *19* (19), 3179-3189.

(20) Katopodes, N. D. Free-Surface Flow. Butterworth-Heinemann, 2019; pp 184-270.

(21) Masoliver, J. Telegraphic Transport Processes and Their Fractional Generalization: A Review and Some Extensions. *Entropy (Basel)* **2021**, *23* (3).

(22) Li, D.; Chen, A.; Zheng, D.; Li, Z.; Na, R.; Cui, F.; Yang, X. Design and Optimization of Interdigital Capacitive Humidity Sensor with Highly Sensitive and Dynamic Response Time. In *Applied Sciences*, 2022; Vol. 12.

(23) Laville, C.; Pellet, C.; N' Kaoua, G. Interdigitated humidity sensors for a portable clinical microsystem. In *1st Annual International IEEE-EMBS Special Topic Conference on Microtechnologies in Medicine and Biology. Proceedings (Cat. No.00EX451)*, 12-14 Oct. 2000, 2000; pp 572-577.
